# Chiral Oxazoline-Triazole-Benzothiazole Molecular Triads: Photoactive Sensors for Enantioselective Carbohydrate Recognition in Solution

**DOI:** 10.1021/jacsau.4c01131

**Published:** 2025-01-09

**Authors:** Natalí P. Debia, Lilian C. Da Luz, Bruno B. de Araújo, Paulo F. B. Gonçalves, Fabiano S. Rodembusch, Diogo S. Lüdtke

**Affiliations:** Instituto de Química, Universidade Federal do Rio Grande do Sul—UFRGS, Av. Bento Gonçalves 9500, 91501-970 Porto Alegre, Rio Grande do Sul, Brazil

**Keywords:** optical sensor, carbohydrate, enantiomer sensing, enantioselectivity, fluorescence

## Abstract

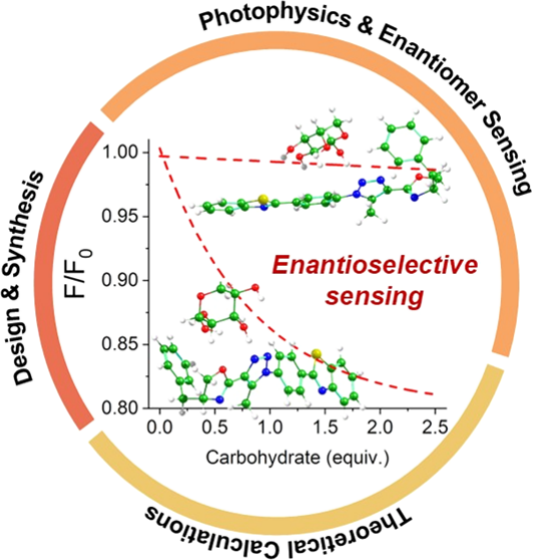

Understanding the mechanism of drug action in biological systems is facilitated by the interactions between small molecules and target chiral biomolecules. In this context, focusing on the enantiomeric recognition of carbohydrates in solution through steady-state fluorescence emission spectroscopy is noteworthy. To this end, we have developed a third generation of chiral optical sensors for carbohydrates, distinct from all of those previously presented, which interact with carbohydrates to form non-covalent probe-analyte interactions. The proposed sensor is based on 2-oxazolines bearing a fluorophoric benzothiazole unit. We evaluated their photophysical properties in the presence of enantiomeric pairs of arabinose, mannose, xylose, and glucose in solution. Our primary findings indicate that the compounds outlined in this study were able to distinguish between enantiomeric pairs in solution, demonstrating good to excellent enantioselectivity through simple intermolecular interactions. To achieve the best enantioselectivity results, theoretical calculations were performed to better understand the observed interactions between the sensors and the analytes.

## Introduction

Heterocyclic systems represent a ubiquitous class of compounds that are key structural elements in various natural and synthetic compounds with biological activity.^1,2^ Beyond their biological applications,^3^ these systems are widely employed in the study of new organic materials and fluorescent probes.^4,5^ A noteworthy core in this context is benzo(thi)azole, which has been studied in a variety of applications in optical sensors, such as ion and pH sensing,^6^ as well as in cancer cell imaging and H_2_S detection.^7,8^ Among its derivatives, the 2-aryl-benzo(thi)azole system is versatile and can be synthesized using various methods.^9−13^

**Figure 1:**
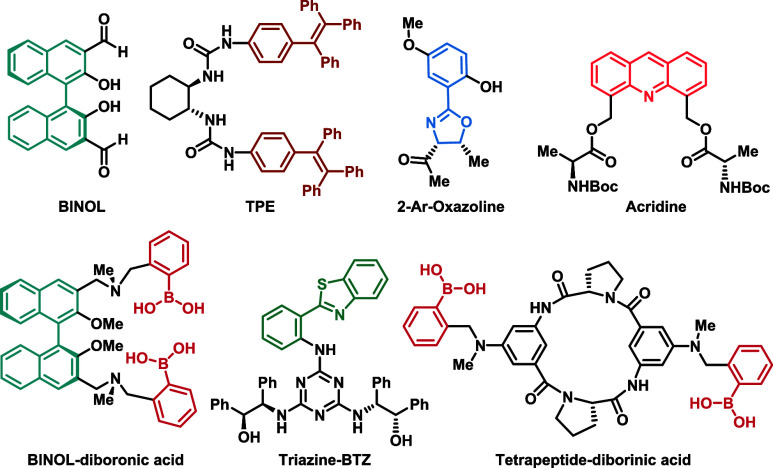
Selected chemical structures of fluorescent optical sensors for the recognition of chiral molecules.

Recently, the development of enantioselective fluorescent sensors for the recognition of chiral molecules has gained prominence in the literature.^14−17^ Fluorescent optical sensors are interesting options for detecting various analytes due to their low cost, high sensitivity, fast response, and good modulation capabilities. Considering the inherent chirality of biomolecules and the role of chiral recognition in the mechanisms of action of chiral molecules within biological systems, research in this field is of significant importance. Enantioselective sensors differentiate enantiomers when the chiral sensor interacts distinctively with each enantiomer of a pair, producing a measurable signal. Among the most commonly used scaffolds in the development of such sensors is 1,1′-bi-2-naphthol (BINOL),^15,18^ (Figure 1), which has been employed in the differentiation of carboxylic acids,^19^ amino acids,^20,21^ amino alcohols,^22,23^ and diamines.^24^ Another well-established core is tetraphenylethylene (TPE), utilized for the enantioselective differentiation of amines and free amino acids.^25−29^ Additionally, other structures with notable applications include the acridine core,^30^ which integrates two *N*-Boc-l-alanine units and has been applied for the recognition of the tartrate anion, and benzothiazole (BTZ), linked to a triazine unit,^31^ containing two amino alcohol units used in the enantiomeric differentiation of carboxylic acids. Specifically, for carbohydrates, numerous studies focus on their identification in solution;^32^ however, these reports most often do not describe systems capable of distinguishing between enantiomers.

There are few reports of fluorescent sensors capable of differentiating between the two enantiomers of a pair in solution. Most of these sensors are based on boronic acids, which react with carbohydrates to form a covalent bond between the sensor and the analyte.^33−35^ The first enantioselective sensor for carbohydrates was BINOL-based and contained two boronic acid units (Figure 1).^33^ A decade later, a cyclic tetrapeptide incorporating two boronic acid units was described.^34^ In this context, our group has been focusing on the development of sensors that interact with analytes through intermolecular forces, leading to differential recognition. Pursuing this goal, we have developed two generations of fluorescent probes capable of achieving this objective.^36,37^ These compounds successfully differentiated the enantiomers of carbohydrate arabinose. Subsequently, Santos et al.^38^ presented the synthesis of oxazolines derived from l-threonine (Figure 1), which were studied for the enantioselective identification of mandelic acid and arabinose.

**Scheme 1:**
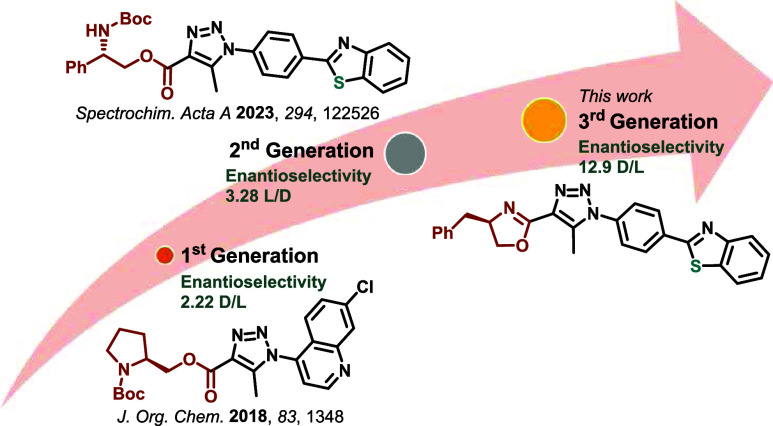
Design of Amino Acid Derivatives as Enantiomeric Fluorescent Probes for Carbohydrates in Solution

Herein, we detail our efforts to improve the enantioselectivity of carbohydrate sensing, which ultimately led to the development of a third generation of probes. Central to the success of our design was the synthesis of new chiral 2-oxazolines embedded with a 2-aryl-benzothiazole core as a fluorescent moiety (Scheme 1). The key to our improved system lies in the introduction of a chiral oxazoline moiety. The oxazoline core is widely used as the chiral unit in the synthesis of ligands for various applications in asymmetric catalysis.^39^ This system has also been applied in the biomedical field for drug delivery and tissue engineering.^40^

## Results and Discussion

### Design and Synthesis of the Probes

**Scheme 2:**
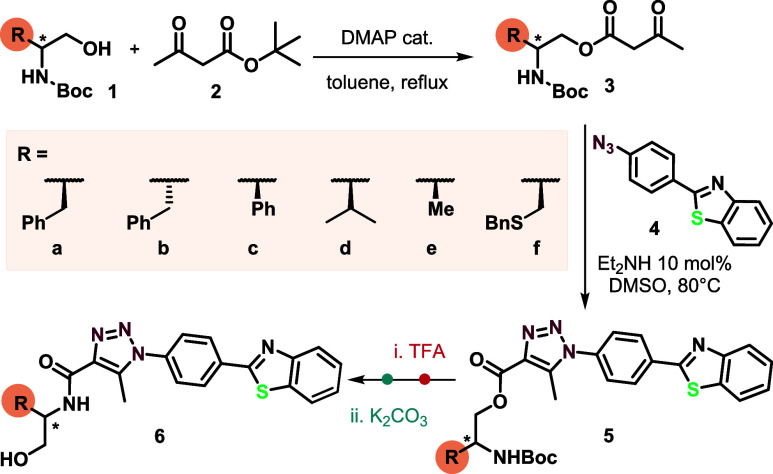
Synthetic Methodology for Obtaining *N*-(2-hydroxyethyl)amides **6a**–**f**

The probes were designed to incorporate a chiral moiety derived from amino acids, such as l- and d-phenylalanine, l-phenylglycine, l-valine, l-alanine, and *S*-benzyl-l-cysteine, along with a fluorescent moiety derived from benzothiazole. The starting materials used to synthesize the desired compounds were prepared according to previous work (Scheme 2).^41^ The synthetic route begins with transesterification of *N*-Boc-amino alcohols **1** using *tert*-butyl acetoacetate (**2**), leading to β-keto esters **3**. These β-keto esters then react with 2-(4′-azidophenyl)-benzothiazole (**4**) through an organocatalytic enamine-azide [3 + 2] cycloaddition, resulting in the 1,2,3-triazole derivatives **5**.

**Scheme 3:**
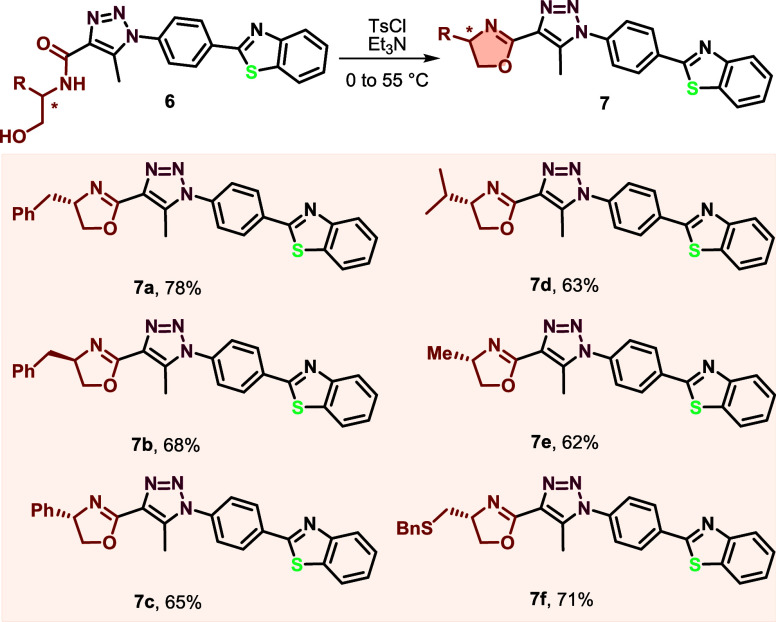
Cyclization for the Synthesis of Sensors **7a**–**f**

The final step involved the *N-*Boc deprotection followed by *O–N* acyl intramolecular migration, resulting in the formation of amino acid *N*-(2-hydroxyethyl)amide derivatives **6**.^42,43^ With these *N*-(2-hydroxyethyl) amides **6a**–**f** in hands, we proceeded to study their cyclization to form 2-oxazolines in the presence of tosyl chloride and triethylamine (Et_3_N). A brief optimization study of this reaction was conducted using amide **6a** as a model compound. Using dry dichloromethane as the solvent at 25 or 35 °C for 30 h yielded 2-oxazoline **7a** with yields of 40 and 53%, respectively. Subsequently, changing the solvent to chloroform and increasing the reaction temperature to 55 °C for 30 h improved the yield to 78%. Applying these optimized reaction conditions to the intramolecular cyclization of the remaining amino acid *N*-(2-hydroxyethyl)amide derivatives enables the synthesis of 2-oxazolines **7a**–**f** in excellent yields ranging from 62 to 78% (Scheme 3). The reaction tolerated various side chains, including benzyl (**7a**–**b**), phenyl (**7c**), isopropyl (**7d**), methyl (**7e**), and a functionalized alkyl chain containing sulfur (**7f**).

### Photophysical Characterization

**Figure 2:**
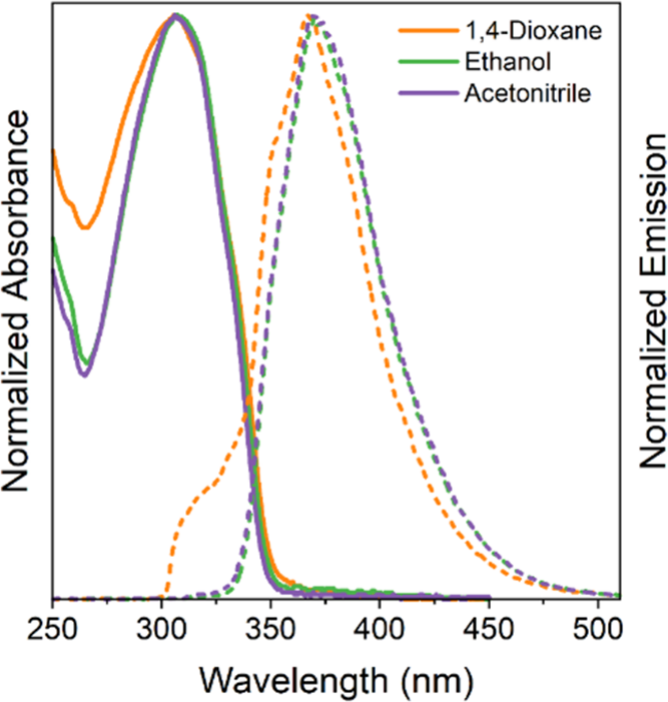
Normalized absorption and steady-state fluorescence emission spectra in solutions of **7b** in different organic solvents (ca. 10^–5^ M, λ_ex_ = 305 nm, ex/em slits 3.0 nm/3.0 nm).

In this study, we conducted a photophysical investigation of compounds **7a**–**f** in three different organic solvents: 1,4-dioxane, ethanol, and acetonitrile. The relevant data from this characterization are summarized in Table 1. The compounds exhibited no significant solvatochromic effects in either their ground or excited states when they were exposed to solvents of varying polarities. Analysis of the UV–vis absorption spectra revealed absorption maxima (λ_abs_) between 306 and 308 nm, attributed to the benzothiazole core (Figure 2). Furthermore, examination of the steady-state fluorescence emission spectra consistently revealed emission maxima (λ_em_) within the range 367–371 nm across all tested solvent polarities. The choice of solvent did not significantly impact these emission values, except for a slight variation observed in the emission curve when 1,4-dioxane was used. The Stokes shift observed for the studied compounds ranged from 61–65 nm, indicating a significant energy loss in the excited state. The normalized absorption and emission spectra of compound **7b** are depicted in Figure 2, providing a clear view of the curve profiles. Similar behaviors were observed in the absorption and emission spectra of other synthesized compounds in the study. For a more detailed analysis, the original UV–vis absorption, fluorescence emission, and excitation spectra for all studied compounds can be found in the Supporting information (Figures S10–S12).

**Table 1:** Photophysical Data of **7a**–**f**^a^

compound	solvent	λ_abs_	λ_em_	Δλ_ST_	ε	*f*_e_	*k*_e_^0^	τ^0^	Φ_FL_
**7a**	1,4-dioxane	306	367	61/5432	2.42	0.74	5.32	1.88	6.6
	ethanol	308	371	63/5513	1.94	0.50	3.56	2.81	4.6
	acetonitrile	306	371	65/5726	1.94	0.52	3.74	2.67	4.1
**7b**	1,4-dioxane	306	368	62/5506	2.62	0.80	5.75	1.74	5.2
	ethanol	308	371	63/5513	2.44	0.65	4.61	2.17	3.8
	acetonitrile	307	370	63/5546	2.17	0.58	4.09	2.45	3.8
**7c**	1,4-dioxane	306	368	62/5506	2.42	0.71	5.10	1.96	5.4
	ethanol	308	371	63/5513	1.89	0.52	3.68	2.72	4.6
	acetonitrile	305	371	66/5833	2.00	0.56	4.02	2.49	4.0
**7d**	1,4-dioxane	306	367	61/5432	2.66	0.73	5.19	1.93	4.4
	ethanol	307	371	64/5619	2.58	0.73	5.22	1.92	3.4
	acetonitrile	306	370	64/5653	2.50	0.67	4.76	2.10	3.4
**7e**	1,4-dioxane	307	368	61/5399	1.98	0.52	3.70	2.71	5.1
	ethanol	308	370	62/5441	1.90	0.45	3.20	3.12	4.1
	acetonitrile	306	370	64/5653	1.90	0.49	3.47	2.88	3.8
**7f**	1,4-dioxane	306	367	61/5432	2.51	0.74	5.27	1.90	5.8
	ethanol	306	371	65/5726	2.39	0.67	4.78	2.09	4.2
	acetonitrile	306	370	64/5653	2.15	0.57	4.11	2.43	4.1

a Where λ_abs_ and λ_em_ are the absorption and emission maxima (nm), respectively; Δλ_ST_ is the Stokes shift (nm/cm^–1^); ε is the molar extinction coefficient (10^4^ M^–1^·cm^–1^); *f*_e_ is the calculated oscillator strength; *k*_e_^0^ is the calculated radiative rate constant (10^8^ s^–1^); τ^0^ is the calculated pure radiative lifetime (ns); and Φ_FL_ is the fluorescence quantum yield (%).

To better characterize the photophysical behavior of compounds **7a**–**f**, we employed the Strickler–Berg relationship, which correlates the absorption intensity with the fluorescence lifetime.

From the UV–vis spectra, the area under the absorption curve—obtained by plotting the molar absorptivity coefficient ε (M^–1^ cm^–1^) against wavenumber *v̅* (cm^–1^)—can be related to the single electron oscillator strength *f*_e_ and the corresponding emission rate constant *k*_e_^0^ through eqs 1 and 2, respectively.^44^ In addition, the pure radiative lifetime τ^0^ is defined as 1/*k*_e_^0^.^45^
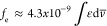
1
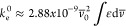
2

Based on the data presented in Table 1, including the molar absorptivity coefficients (ε = (1.89–2.66) × 10^4^ M^–1^ cm^–1^) and the calculated radiative rate constants (*k*_e_^0^ = (3.20–5.75) × 10^8^ s^–1^), we concluded that all the compounds exhibit electronic transitions that are fully allowed by spin and symmetry selection rules, attributed to π–π* transitions. The structural rigidity in the chromophoric units of these compounds resulted in relatively high oscillator strengths (*f*_e_ = 0.45–0.80). An almost constant pure radiative lifetime (τ^0^ = 1.74–3.12 ns) was observed for each compound across different solvents, indicating that these compounds populate the same excited state. The fluorescence emission spectra of all the compounds were obtained at two excitation wavelengths: the absorption maximum at 305 nm and the shoulder at 330 nm. Analysis of the spectra (Figure S11) shows that compounds **7a**–**f** follow Kasha’s rule, presenting the same emission maxima at both excitation wavelengths. From the spectra acquired at λ_ex_ = 305 nm, the fluorescence quantum yield (Φ_FL_) values ranged from 3.36 to 6.57%. All the compounds showed Φ_FL_ values slightly higher in 1,4-dioxane (4.40–6.57%) compared to similar values in ethanol and acetonitrile (3.36–4.58%).

### Enantiomer Sensing in Solution

**Figure 3:**
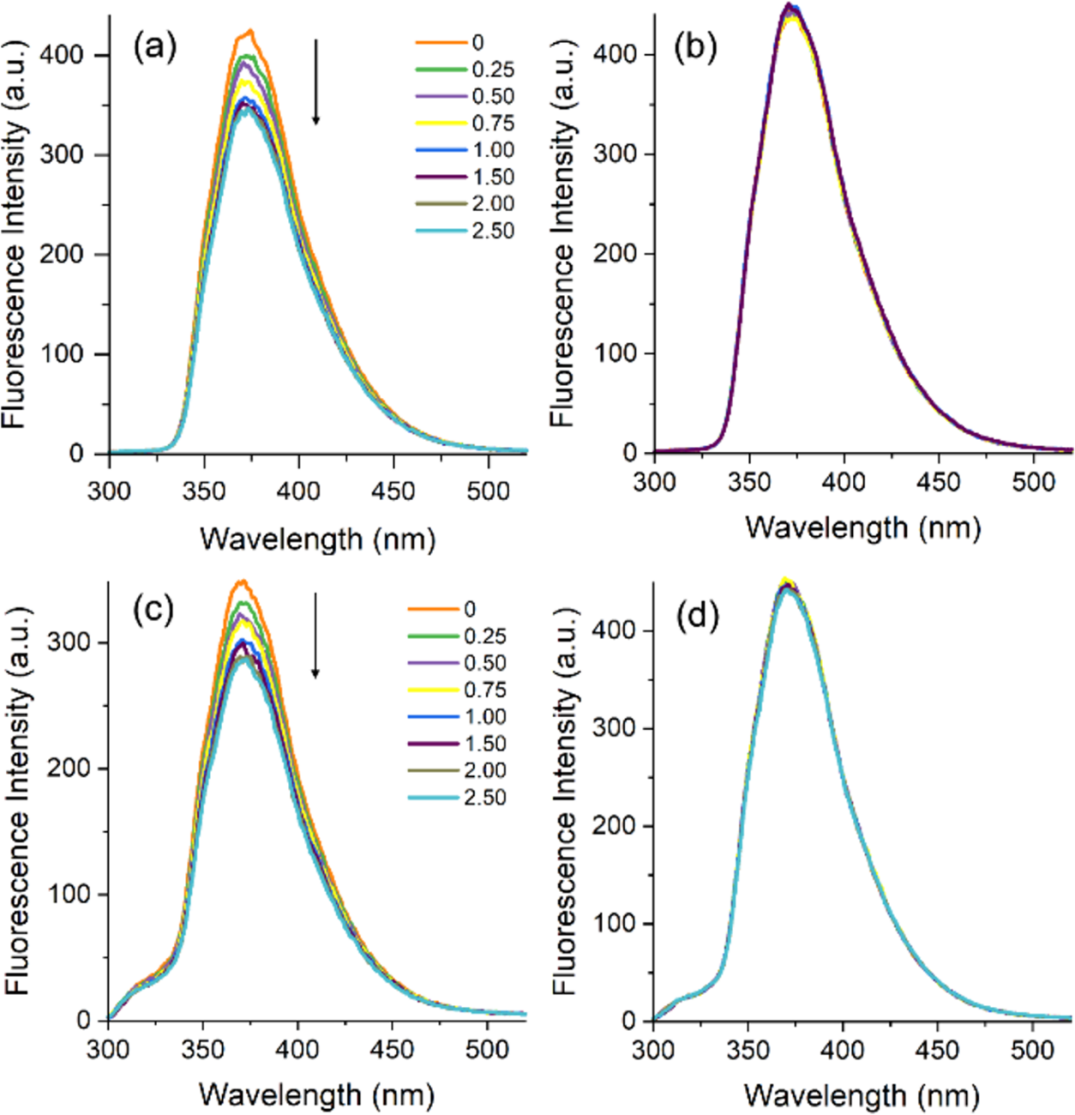
Fluorescence emission spectra of **7b** (ca. 10^–5^ M) in the presence of different amounts (0–2.50 equiv) of (a) d- and (b) l-arabinose and (c) d- and (d) l-mannose enantiomers. (λ_ex_ = 305 nm, ex./em. slits 3.0 nm/3.0 nm).

A sensing investigation was conducted on compounds **7a**–**f** in an acetonitrile solution. Acetonitrile was selected due to its high solubility for these compounds and minimal interference in the measurements. Carbohydrate enantiomers were dissolved in DMSO because of their limited solubility in acetonitrile. Fluorometric titrations were performed with varying amounts (0–2.50 equiv) of enantiomers added. The working concentration for d- and l-arabinose solutions was 1.4 mM, and those for d- and l-mannose solutions were 1.5 mM. After each addition, UV–vis absorption and fluorescence emission spectra were recorded (Figures S13–S16 and S18–S21). Generally, no significant changes were observed in the UV–vis absorption spectra of the compounds upon the addition of the enantiomer solutions. However, for compound **7b**, an increase in absorption was noted upon the addition of the d-enantiomers of arabinose and mannose. In the fluorescence emission spectra, enantiomeric differentiation was observed for compounds **7b**, **7c**, and **7f** with arabinose and for compounds **7b**, **7d**, and **7f** with mannose. Figure 3 shows the fluorescence emission spectrum of **7b** after the addition of each enantiomer. A distinct decrease in emission intensity is evident upon interaction with the d-enantiomers of both carbohydrate pairs studied. In this case, the excited state of **7b** appears to be significantly affected by the presence of d-arabinose and d-mannose. Conversely, the excited state of **7c** is influenced by l-arabinose (Figure S15), and that of **7d** is affected by l-mannose (Figure S21). Additionally, the excited state of compound **7f** is impacted by both l-arabinose and l-mannose (Figures S16 and S21). By analyzing the fluorescence emission curves obtained after each successive addition, we constructed graphs relating the intensity ratio (*F*/*F*_0_) to the amount of each equivalent added (Figures S17 and S22).

**Figure 4:**
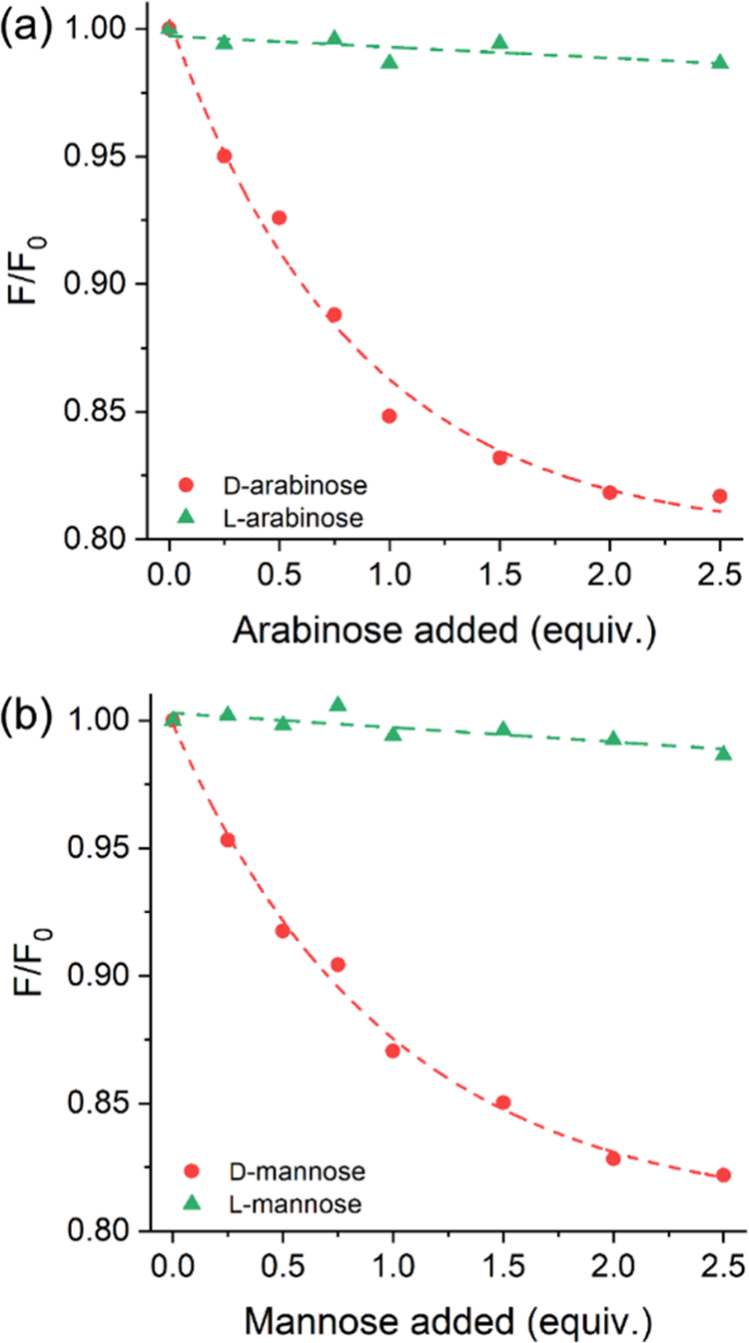
Intensities ratio of the fluorescence emission of **7b** (2.43 × 10^–5^ M) with different amounts of (a) d- and l-arabinose and (b) d- and l-mannose enantiomers.

Figure 4 depicts the graph for compound **7b**, where a significant difference in the interaction between this compound and the enantiomers is evident in both cases studied. Notably, for the interaction of **7b** with l-arabinose and l-mannose, the *F*/*F*_0_ ratio remained almost constant after each addition. However, when interacting with d-arabinose and d-mannose, an exponential decrease in the *F*/*F*_0_ ratio was observed with each added equivalent. To better evaluate the interactions between the compound and the enantiomers, we calculated the fluorescence sensitivity (*I*_D_/*I*_0_ and *I*_L_/*I*_0_) and enantioselectivity (*I*_D_–*I*_0_/*I*_L_–*I*_0_ and *I*_L_–*I*_0_/*I*_D_–*I*_0_) from the fluorescence emission curves. The relevant data for the recognition of arabinose and mannose pairs are summarized in Table 2. Enantioselectivity values close to unity indicate a lack of significant differentiation among the enantiomers of a given pair in solution. Analysis of the calculated enantioselectivities revealed amino acid derivatives **7b** (from d-phenylalanine), **7c** (from l-phenylglycine), **7d** (from l-valine), and **7f** (from *S*-benzyl-l-cysteine). Among these, compound **7b** showed the highest selectivity for d-arabinose (12.9) and d-mannose (10.3). In contrast, compounds **7c** and **7d** presented higher selectivities for l-arabinose (2.54) and l-mannose (5.23), respectively. Compound **7f** showed selectivity for both l-arabinose (2.01) and l-mannose (2.55).

**Table 2:** Fluorescence Sensitivity and Enantioselectivity at λ_ex_ = 305 nm of Sensors **7a**–**f** Using d- and l-Arabinose and d- and l-Mannose as Analytes

	arabinose				mannose			
	fluorescence sensitivity				fluorescence sensitivity			
compound	*I*_D_/*I*_0_	*I*_L_/*I*_0_	enantioselectivity*		*I*_D_/*I*_0_	*I*_L_/*I*_0_	enantioselectivity*	
**7a**	0.95	0.94	1.08	L/D	0.96	0.97	1.28	D/L
**7b**	0.82	0.99	12.9	D/L	0.82	0.99	10.3	D/L
**7c**	0.96	0.89	2.54	L/D	0.98	0.99	1.82	D/L
**7d**	0.98	0.97	1.28	L/D	0.98	0.90	5.23	L/D
**7e**	0.97	0.96	1.25	L/D	0.98	0.98	1.18	D/L
**7f**	0.94	0.88	2.01	L/D	0.98	0.95	2.55	L/D

In a previous work, enantioselective D/L values of 2.22 and 1.25 were obtained for *N*-Boc-l-proline and *N*-H-l-proline derivatives, respectively, with the arabinose pair.^37^ In another study, an *N*-Boc-l-phenylglycine derivative showed an enantioselective L/D value of 3.28 for the arabinose pair. Moreover, amino acid *N*-(2-hydroxyethyl)amide derivatives presented enantioselectivities of 2.34 D/L (from l-valine), 2.29 D/L (from l-phenylalanine), and 2.16 L/D (from d-phenylalanine) for the arabinose pair.^36^

In both cases, differentiation occurs through intermolecular forces, distinguishing our approach from other studies in the literature that involve covalent bond formation.^33−35^ The present work describes a new series of amino acid derivatives capable of differentiating arabinose and mannose enantiomers in solution, primarily due to hydrogen bonding interactions. Compound **7b** showed excellent enantioselectivity toward both enantiomer pairs studied.

Compounds **7b** and **7f**, which showed the best results in identifying enantiomer pairs, were also investigated with glucose and xylose enantiomers (Figures S23–S25). The relevant results are listed in Table 3. Analysis of the calculated enantioselectivities revealed that compound **7b** showed a higher selectivity for d-glucose (7.30). In contrast, compound **7f** presented similar selectivity for l-glucose (2.00) and l-xylose (2.58).

**Table 3:** Fluorescence Sensitivity and Enantioselectivity at λ_ex_ = 305 nm of Sensors **7b** and **7f** Using d- and l-Glucose and d- and l-Xylose as Analytes

	glucose				xylose			
	fluorescence sensitivity				fluorescence sensitivity			
compound	*I*_D_/*I*_0_	*I*_L_/*I*_0_	enantioselectivity*		*I*_D_/*I*_0_	*I*_L_/*I*_0_	enantioselectivity*	
**7b**	0.63	0.95	7.30	D/L	0.87	0.96	2.26	D/L
**7f**	0.93	0.86	2.00	L/D	0.94	0.87	2.58	L/D

To gain deeper insights into the interaction mechanism between the sensors and chiral analytes, we employed time-resolved spectroscopy to study enantiomer pairs showing the most significant interaction differences with the sensor. Accordingly, compound **7b** was selected as a model compound, and its fluorescence lifetime was measured in solution both in the absence and presence of l- and d-arabinose. The original spectra and results are available in the Supporting Information (Figures S26–S28 and Table S2). The sensor exhibited very similar fluorescence lifetime in its pure form (∼0.24 ns) and in the presence of 2.5 equiv of l-arabinose (∼0.23 ns) or d-arabinose (∼0.23 ns). These results indicate that, regardless of the enantiomer present, the sensor’s lifetime remains nearly unchanged. This suggests that the observed fluorescence quenching is static in nature,^46^ indicating that the interaction between the sensor and the analyte, although weak, occurs in the ground state, thus ruling out collisional deactivation in the excited state. These findings support the hypothesis of a weak but sufficiently effective interaction that allows a nonradiative deactivation pathway for the sensor’s excited state.

### Theoretical Calculations

**Figure 5:**
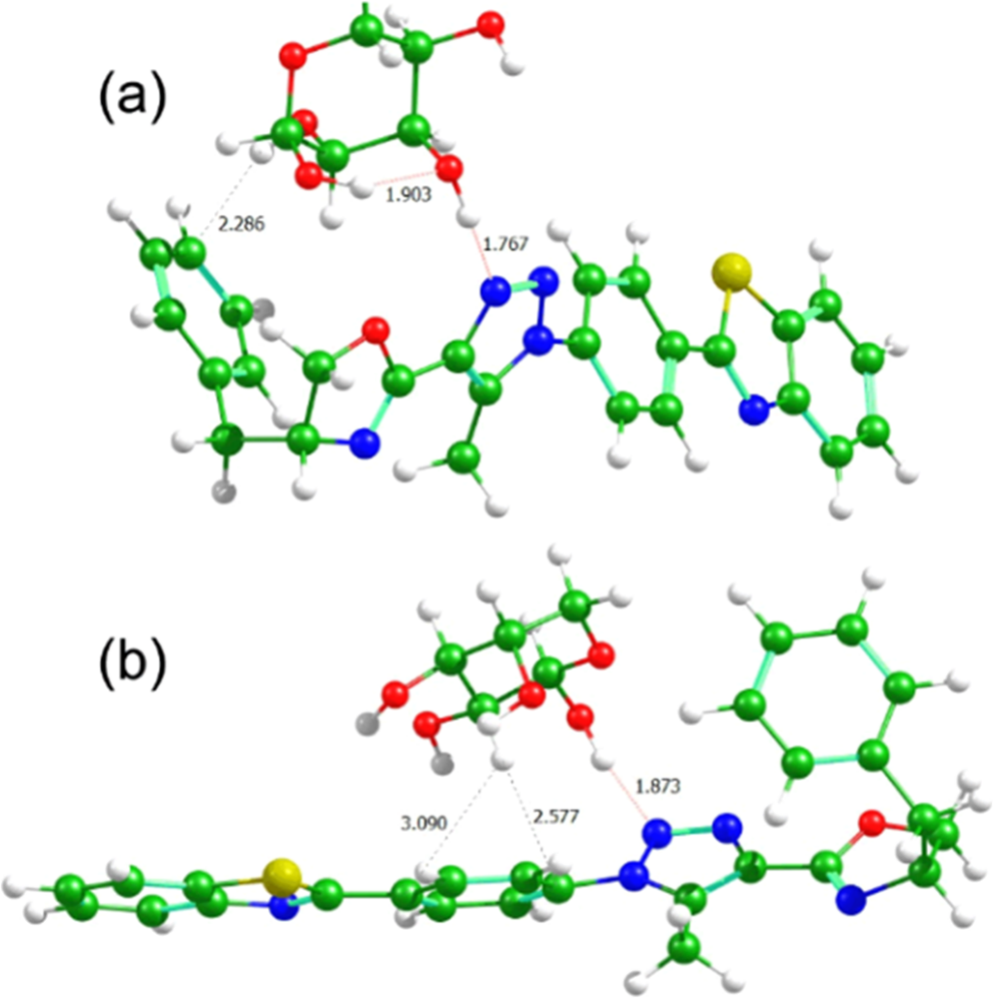
Interaction systems of **7b** with (a) d-arabinose and (b) l-arabinose. The distances are shown in angstroms for the hydrogen bonds and for the potential CH–π interactions (not observed).

**Figure 6:**
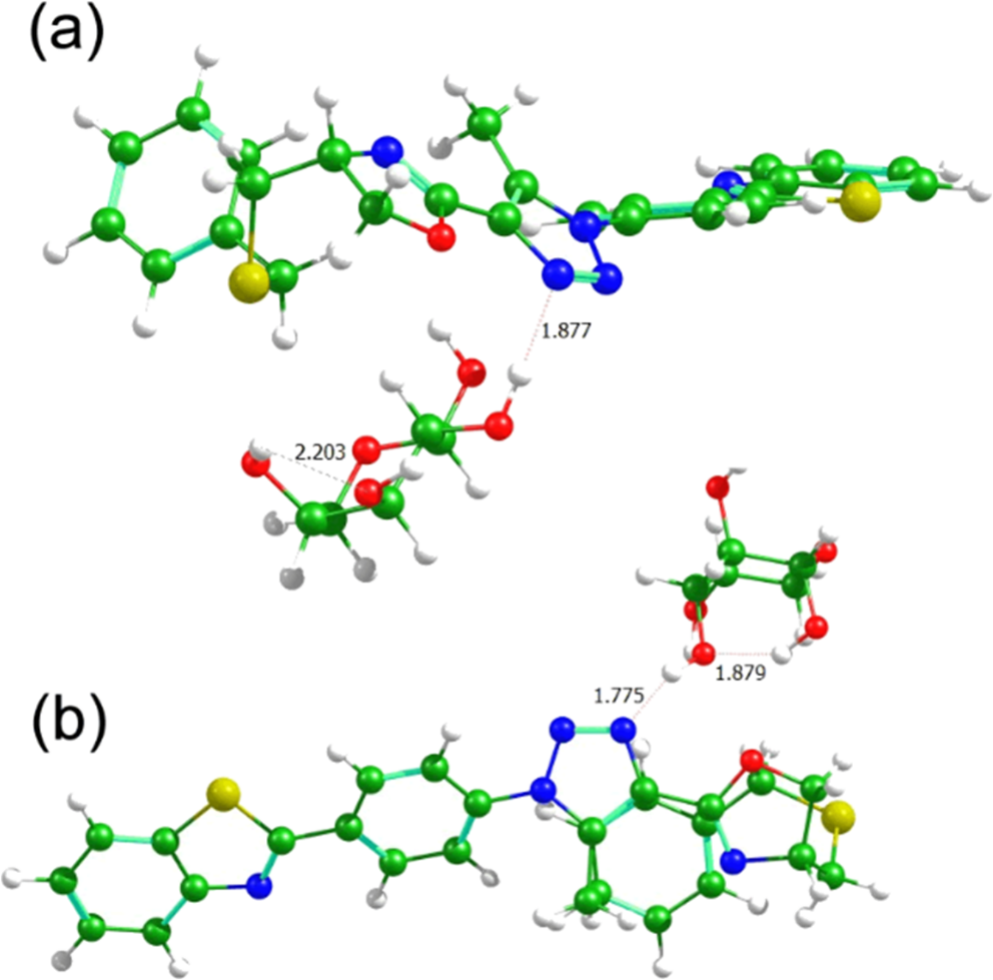
Interaction systems of **7f** with (a) d-arabinose and (b) l-arabinose. The distances are shown in angstroms.

To better understand the experimental findings regarding sensor-enantiomer interactions, we performed density functional theory (DFT) calculations. We used compounds **7b** and **7f** as models in combination with the d- and l-enantiomers of mannose and arabinose. The differences in interaction energies between the L and D forms of arabinose and mannose with the model compounds can generally be attributed to their spatial orientation and the surrounding chemical environment, as illustrated in Figures 5 and 6. The relevant data are summarized in Table 4.

**Table 4:** Interaction Energies in kcal·mol^–1^ of Compounds **7b** and **7f** with d/l-Mannose and d/l-Arabinose Enantiomers

	arabinose			mannose		
compound	*D*	*L*	Δ*E*_DL_	*D*	*L*	Δ*E*_DL_
**7b**	–11.77	–9.32	2.45	–7.43	–7.24	0.19
**7f**	–7.06	–9.14	2.08	–8.73	–8.78	0.05

Regarding compound **7b**, the proximity of the d-enantiomers (d-arabinose and d-mannose) to the phenyl group of the dihydroxazole ring facilitates stabilizing van der Waals interactions, as evidenced by the interaction between **7b** and d-arabinose, resulting in lower interaction energies compared to their l-form counterparts (Figures 5 and S29). The interaction study using **7b** and mannose is presented in the Supporting Information (Figure S30). Three types of noncovalent interactions were analyzed: the common hydrogen bonding and van der Waals interactions, along with the more exotic CH/π interactions. The latter plays a distinctive role in stabilizing carbohydrate/aromatic complexes. Often described as a nonconventional hydrogen bond between CH groups and aromatic rings, CH/π interactions have recently gained attention in the literature due to their significance in biomolecular complexes, organocatalysis, and related fields.^47,48^ Hydrogen bonds are particularly important for their strong stabilization effects, which facilitate the formation of carbohydrate/aromatic complexes. Additionally, they needed to displace water molecules and create hydrophobic cavities that could effectively accommodate substrates.^49^

A weak CH/π interaction was observed between these molecules; however, its effect is limited by the relatively large distance and the unfavorable alignment between the interacting systems. The spatial separation is at the upper limit of the typical range for CH/π interactions,^48^ and the geometric orientation does not favor efficient overlap of the interacting components. The only significant case was the interaction between molecule **7b** and l-arabinose, with the relevant distances shown in Figure S31. Despite these suboptimal distances, a weak CH/π interaction is still observed but it is not strong enough to significantly stabilize the system. These findings are consistent across all of the analyzed configurations.

Specifically, the positioning of the d-enantiomers allows them to engage more favorably with the phenyl group, thereby exerting a stronger stabilization effect. In contrast, the l forms of the carbohydrates are primarily situated near the phenyl ring adjacent to the 1,2,3-triazole group, leading to less favorable interactions and consequently lower interaction energies. Overall, this difference in interaction energies (Δ*E*_DL_) between the D and L forms of arabinose (2.45 kcal mol^–1^) and mannose (0.19 kcal mol^–1^) with **7b** can be attributed to the specific spatial arrangement of the carbohydrates and their interactions with the surrounding chemical groups.

Figures 6 and S32 present the interaction study of compound **7f** and arabinose. Unlike compound **7b**, derivative **7f** exhibited lower interaction energies with l-enantiomers (Table 4). An interaction study involving this compound and mannose is provided (Figure S33). Notably, in this case, the orientations of the carbohydrates account for the reduced enantioselectivity of **7f** toward the D and L forms of arabinose and mannose (Δ*E*_DL_: 2.08 kcal mol^–1^ for arabinose and 0.05 kcal mol^–1^ for mannose), highlighting the minimal discrimination. Here, the phenyl group of dihydroxazole could not interact as effectively with the carbohydrates, resulting in similar interactions for both enantiomers. These findings underline the subtle structural differences between the derivatives and their impact on carbohydrates recognition. Therefore, the spatial arrangement of d carbohydrates is more favorable for the formation of hydrogen bonds and other stabilizing interactions. In contrast, the l-carbohydrates—specifically the **7f** compounds—are positioned less favorably for energetically significant interactions such as hydrogen bonding.

## Conclusions

Herein, we describe the synthesis of a novel series of amino-acid-derived 2-oxazolines incorporating a benzothiazole unit linked by a 1,2,3-triazole moiety. Compounds **7a**–**f** were synthesized via intramolecular cyclization of *N*-(2-hydroxyethyl)amides, achieving good yields ranging from 62 to 78%. Photophysical characterization using UV–vis absorption and fluorescence emission spectroscopy revealed absorption maxima at approximately 305 nm and emission maxima at approximately 370 nm. Furthermore, we investigated compounds **7a**–**f** as optical sensors for fluorescent enantioselective differentiation of carbohydrate enantiomers in solution. Our results demonstrate distinct optical responses of the compounds in the presence of the enantiomers. Notably, compound **7b** exhibited calculated enantioselectivity values of 12.9 D/L for arabinose and 10.3 D/L for mannose, which are higher than those previously reported. In this work, the observed response is mainly attributed to weak interaction forces, such as hydrogen bonds. These findings expand the repertoire of molecules available for the fluorescent enantiomeric differentiation of d- and l-enantiomers of carbohydrates in solution.

## Methods

### General Procedure for the Synthesis of Oxazolines **7a**–**f**

In a round-bottom flask under an argon atmosphere, the appropriate *N*-(2-hydroxyethyl)amide **6** (1.0 equiv, 0.15 mmol), Et_3_N (6.0 equiv, 0.90 mmol), and dry CHCl_3_ (2 mL) were added. The system was cooled to 0 °C, TsCl (2.0 equiv, 0.30 mmol) was added, and the solution was stirred for 10 min. Next, the ice bath was removed, and after the solution returned to room temperature, the reaction mixture was heated at 55 °C for 24 h. At the end of the reaction, the mixture was diluted with 5 mL of CH_2_Cl_2_ and washed with 1.0 M HCl (5 mL), saturated NaHCO_3_ (5 mL), and saturated NaCl (5 mL). The organic phase was dried with MgSO_4_, filtered, and reduced under a vacuum. The crude material was purified by flash column chromatography, typically eluting with a mixture of CH_2_Cl_2_/MeOH/NH_2_OH, 99.0:0.5:0.5 → 97.5:2.0:0.5.

### Enantiomer Sensing in Solution

Compounds (**7a**–**f**) were dissolved in acetonitrile, resulting in working concentration solutions of 33–48 mM. Varying amounts (0–2.50 equiv) of each enantiomer in DMSO solution were then added to these solutions. The working concentrations for arabinose enantiomers were 1.5 mM, and those for mannose enantiomers were 1.4 mM. After each addition to the compound solution, the mixture was allowed to equilibrate for 30 s before the respective UV–vis and steady-state fluorescence spectra were acquired. Fluorometric titration was performed using an excitation wavelength of 305 nm with excitation/emission slits of 3.0/3.0 nm. All experiments were carried out at 25 °C. The emission maxima from the fluorescence curves were obtained using the function Peak Analyzer function from OriginPro 2021, which provides several methods for automatic peak detection.

### Theoretical Calculations

Compounds **7b** and **7f**, which presented better results for sensing, were optimized using the PBE1PBE/def2-SVPD level of theory. To quantify the strength of these interactions, energy evaluations were performed at the PBE1PBE/def2-TZVPPD level of theory. A combination of double-ζ and triple-ζ basis sets was employed to achieve reasonable optimized geometries and accurate energies at a practical computational cost.^50^ The PBE1PBE functional was selected due to its successful application in our previous work,^37^ along with the addition of Grimme’s dispersion model with Becke–Johnson damping (GD3BJ) to correctly model weak interactions between molecules.^51,52^ Since the experiments were conducted in acetonitrile, this solvent was implicitly simulated using the SMD model.^53^ Density functional theory calculations were carried out using Gaussian 16 rev. A.03 package and Multiwfv.^54−56^

## Supporting Information Available

Analytical data; copies of NMR spectra; and additional photophysical and computational data (PDF)
