# Supplementary material for: Chiral Oxazoline-Triazole-Benzothiazole Molecular Triads: Photoactive Sensors for Enantioselective Carbohydrate Recognition in Solution
Source: JACS Au. 2025 Jan 9;5(1):353–62. doi: 10.1021/jacsau.4c01131 (PMC11775706; doi:10.1021/jacsau.4c01131)
Supplement: Supplementary file 1 — au4c01131_si_001.pdf [file au4c01131_si_001.pdf]

Supporting Information

Chiral Oxazoline-Triazole-Benzothiazole Molecular Triads. Photoactive Sensors for Enantioselective Carbohydrate Recognition in Solution

Natalí P. Debia, Lilian C. da Luz, Bruno B. de Araújo, Paulo F. B. Gonçalves,* Fabiano S. Rodembusch,* Diogo S. Lüdtke*

Instituto de Química, Universidade Federal do Rio Grande do Sul – UFRGS
Av. Bento Gonçalves 9500, 91501-970 Porto Alegre, RS, Brazil.

Contents

1 General information 2
2 General procedure for the synthesis of oxazolines 7a-f 3
3 NMR spectra 6
4 Absorption, emission and excitation spectra 15
5 Additional data for arabinose interaction study 18
6 Additional data for mannose interaction study 23
7 Additional data for glucose and xylose interaction studies 28
8 Additional time-resolved fluorescence spectroscopy data 31
9 Additional computational data 37

1 General information

Air- and moisture-sensitive reactions were conducted in flame- or oven-dried glassware equipped with tightly fitted rubber septa and under a positive pressure of dry argon. Reagents and solvents were handled by using standard syringe techniques. Temperatures above room temperature were maintained by the use of a mineral oil bath heated on a hot plate. Column chromatography was performed using silica gel (230−400 mesh) following the methods described by Still.[i] TLC was performed using supported silica gel GF254 (0.25 mm thickness). For visualization, TLC plates were placed under ultraviolet light and treated with acid vanillin followed by heating. ^{1}H NMR spectra were recorded either with a Varian 400 MHz or a Bruker 400 MHz instrument in $CDCl_3$ solutions. Chemical shifts are reported in ppm, referenced to the solvent peak of residual $CHCl_3$ or TMS as a reference. The following data are reported: chemical shift (δ), multiplicity, coupling constant (J) in Hz, and integrated intensity. Abbreviations to denote the multiplicity of a particular signal are s (singlet), d (doublet), t (triplet), dd (double doublet), m (multiplet), q (quartet), and br (broad singlet). ^{13}C NMR spectra were recorded at 100 MHz in $CDCl_3$ solutions, and the chemical shifts are reported in ppm, referenced to the solvent peak $CDCl_3$. ESI-QTOF-MS measurements were performed in positive ion mode (*m/z* 50−2000 range). Melting points were obtained on a Buchi M-565. Optical rotations were measured using a JASCO P-200 digital polarimeter with 10 mm cells and are reported as $[\alpha]_{20}^{D}$, concentration (g/100 mL), and solvent. Spectroscopic grade solvents were used in the photophysical study. The UV−vis absorption spectra in solution were acquired on a Shimadzu UV-2450 spectrophotometer (with a data interval of 1.0 nm), and the steady-state fluorescence spectra were measured on a Shimadzu spectrofluorometer model RF-5301PC. Quinine sulfate (Riedel) in 0.5 M H_2SO_4 (Φ_{FL}=0.55) was used as the quantum yield standard.[ii] The fluorescence time decay curves were obtained in acetonitrile using an EasyLife V spectrophotometer (OBB), with Ludox® as the instrument response function (IRF). A cutoff filter at 420 nm was used to separate the laser scattering from the studied samples. A pulsed LED light source operating at 340 nm was used for these experiments, which were all conducted at room temperature (25 °C). The analysis of the fluorescence decay curves was carried out via EasyLife V software from OBB. To fit the decay to a sum of exponentials, a nonlinear least squares method was utilized. The fit quality was assessed on the basis of the value of χ^2, residuals, and the autocorrelation function.

2 General procedure for the synthesis of oxazolines 7a-f

In a round bottom flask under an argon atmosphere, the appropriate *N*-(2-hydroxyethyl)amide **6** (1.0 equiv., 0.15 mmol), Et_3N (6.0 equiv., 0.90 mmol), and dry $CHCl_3$ (2 mL) were added. The system was cooled to 0 °C, TsCl (2.0 equiv., 0.30 mmol) was added, and the solution was stirred for 10 min. Next, the iced bath was removed, and after the solution returned to room temperature, the reaction mixture was heated at 55 °C for 24 h. At the end of the reaction, the mixture was diluted with 5 mL of CH_2Cl_2 and washed with 1.0 M HCl (5 mL), saturated $NaHCO_3$ (5 mL), and saturated NaCl (5 mL). The organic phase was dried with $MgSO_4$, filtered, and reduced under vacuum. The crude material was purified by flash column chromatography, typically eluting with a mixture of CH_2Cl_2/MeOH/NH_2OH, 99.0:0.5:0.5 → 97.5:2.0:0.5.

(S)-2-(1-(4-(benzo[d]thiazol-2-yl)phenyl)-5-methyl-1H-1,2,3-triazol-4-yl)-4-benzyl-4,5-dihydrooxazole (7a). A 78% yield of the Beige solid was obtained (52.7 mg, 0.12 mmol). M.p.: 204–209 °C. $[\alpha]_{20}^{D}$= -5.29 (*c* 1.04, CH_2Cl_2). ^1H NMR (400 MHz, $CDCl_3$) δ 8.29 (d, *J* = 8.6 Hz, 2H), 8.14–8.08 (m, 1H), 7.97–7.91 (m, 1H), 7.64 (d, *J* = 8.5 Hz, 2H), 7.57–7.50 (m, 1H), 7.47–7.40 (m, 1H), 7.35–7.20 (m, 5H), 4.69–4.59 (m, 1H), 4.46–4.37 (m, 1H), 4.20 (dd, *J* = 8.6, 7.4 Hz, 1H), 3.25 (dd, *J* = 13.7, 5.3 Hz, 1H), 2.77 (dd, *J* = 13.7, 8.7 Hz, 1H), 2.68 (s, 3H). ^{13}C NMR (100 MHz, $CDCl_3$) δ 165.8, 157.8, 154.0, 137.8, 137.3, 136.1, 135.2, 135.0, 134.9, 129.2, 128.6, 128.5, 126.6, 126.5, 125.7, 125.5, 123.5, 121.7, 71.6, 67.9, 41.8, 10.3. HRMS-ESI calculated for $[C_{26}H_{21}N_5O_S+H]^+$: 452.1540, found 452.1550.

(R)-2-(1-(4-(benzo[d]thiazol-2-yl)phenyl)-5-methyl-1H-1,2,3-triazol-4-yl)-4-benzyl-4,5-dihydrooxazole (7b). The Beige solid was obtained in 68% yield (46.1 mg, 0.10 mmol). M.p.: 204–209 °C. $[\alpha]_{20}^{D}$= 5.29 (*c* 0.76, CH_2Cl_2). ^1H NMR (400 MHz, $CDCl_3$) δ 8.31 (d, *J* = 8.6 Hz, 2H), 8.15 – 8.10 (m, 1H), 7.99 – 7.94 (m, 1H), 7.65 (d, *J* = 8.6 Hz, 2H), 7.57 – 7.52 (m, 1H), 7.48 – 7.42 (m, 1H), 7.37 – 7.21 (m, 5H), 4.70 – 4.60 (m, 1H), 4.42 (d, *J* = 9.4, 8.5 Hz, 1H), 4.21 (dd, *J* = 8.5, 7.4 Hz, 1H), 3.26 (dd, *J* = 13.7, 5.4 Hz, 1H), 2.77 (dd, *J* = 13.7, 8.7 Hz, 1H), 2.69 (s, 3H). ^{13}C NMR (100 MHz, $CDCl_3$) δ 165.8, 157.89, 154.1, 137.8, 137.4, 136.2, 135.2, 135.0, 134.9, 129.2, 128.6, 128.5, 126.7, 126.5, 125.8, 125.5, 123.6, 121.7, 71.6, 68.0, 41.8, 10.3. HRMS-ESI calculated for $[C_{26}H_{21}N_5OS+H]^+$: 452.1540, found 452.1537.

(S)-2-(1-(4-(benzo[d]thiazol-2-yl)phenyl)-5-methyl-1H-1,2,3-triazol-4-yl)-4-phenyl-4,5-dihydrooxazole (7c). A 65% yield of beige solid was obtained (42.4 mg, 0.10 mmol). M.p.: 108 – 184 °C. $[\alpha]_{20}^{D}$= 29.97 (*c* 1.00, CH_2Cl_2). ^1H NMR (400 MHz, $CDCl_3$) δ 8.31 (d, *J* = 8.6 Hz, 2H), 8.15 – 8.10 (m, 1H), 7.97 – 7.93 (m, 1H), 7.66 (d, *J* = 8.6 Hz, 2H), 7.57 – 7.51 (m, 1H), 7.47 – 7.42 (m, 1H), 7.41 – 7.28 (m, 5H), 5.46 (dd, *J* = 10.1, 8.2 Hz, 1H), 4.86 (dd, *J* = 10.2, 8.4 Hz, 1H), 4.36 – 4.30 (m, 1H), 2.72 (s, 3H). ^{13}C NMR (100 MHz, $CDCl_3$) δ 165.8, 158.7, 154.0, 142.1, 137.3, 136.6, 135.2, 135.0, 134.9, 128.8, 128.7, 127.7, 126.7, 125.8, 125.6, 123.5, 121.7, 74.4, 70.1, 10.4. HRMS-ESI calculated for $[C_{25}H_{19}N_5OS+H]^+$: 438.1383, found 438.1376.

(S)-2-(1-(4-(benzo[d]thiazol-2-yl)phenyl)-5-methyl-1H-1,2,3-triazol-4-yl)-4-isopropyl-4,5-dihydrooxazole (7d). A 63% yield of the Beige solid was obtained (38.3 mg, 0.09 mmol). M.p.: 189 – 194 °C. $[\alpha]_{20}^{D}$= -13.30 (*c* 0.92, CH_2Cl_2). ^1H NMR (400 MHz, $CDCl_3$) δ 8.30 (d, *J* = 8.6 Hz, 2H), 8.14 – 8.10 (m, 1H), 7.98 – 7.93 (m, 1H), 7.65 (d, *J* = 8.6 Hz, 2H), 7.58 – 7.52 (m, 1H), 7.48 – 7.41 (m, 1H), 4.54 – 4.42 (m, 1H), 4.22 – 4.12 (m, 2H), 2.68 (s, 3H), 1.87 (h, *J* = 6.6 Hz, 1H), 1.06 (d, *J* = 6.7 Hz, 3H), 0.97 (d, *J* = 6.7 Hz, 3H). ^{13}C NMR (100 MHz, $CDCl_3$) δ 165.8, 157.2, 154.0, 137.4, 136.0, 135.2, 135.1, 134.9, 128.6, 126.6, 125.7, 125.5, 123.5, 121.7, 72.7, 70.0, 32.9, 18.9, 18.3, 10.2. HRMS-ESI calculated for $[C_{22}H_{21}N_5OS+H]^+$: 404.1540, found 404.1546.

(S)-2-(1-(4-(benzo[d]thiazol-2-yl)phenyl)-5-methyl-1H-1,2,3-triazol-4-yl)-4-methyl-4,5-dihydrooxazole (7e). The yield of the beige solid was 62% (35.2 mg, 0.09 mmol). M.p.: 199 – 203 °C. $[\alpha]_{20}^{D}$= -24.37 (*c* 1.06, CH_2Cl_2). ^1H NMR (400 MHz, $CDCl_3$) δ 8.30 (d, *J* = 8.6 Hz, 2H), 8.14 – 8.10 (m, 1H), 7.97 – 7.93 (m, 1H), 7.64 (d, *J* = 8.6 Hz, 2H), 7.57 – 7.51 (m, 1H), 7.48 – 7.41 (m, 1H), 4.57 (dd, *J* = 9.4, 8.1 Hz, 1H), 4.50 – 4.37 (m, 1H), 4.06 – 3.97 (m, 1H), 2.67 (s, 3H), 1.39 (d, *J* = 6.6 Hz, 3H). ^{13}C NMR (100 MHz, $CDCl_3$) δ 165.8, 157.3, 154.0, 137.4, 136.0, 135.2, 135.1, 134.9, 128.6, 126.6, 125.7, 125.5, 123.5, 121.7, 73.7, 62.1, 21.5, 10.2. HMRS-ESI calculated for $[C_{20}H_{17}N_5OS+H]^+$: 376.1227, found 376.1237.

(R)-2-(1-(4-(benzo[d]thiazol-2-yl)phenyl)-5-methyl-1H-1,2,3-triazol-4-yl)-4-((benzylthio)methyl)-4,5-dihydrooxazole 7f. A 71% yield of the beige solid was obtained (42.3 mg, 0.08 mmol). M.p.: 151 – 155 °C. $[\alpha]_{20}^{D}$= 24.62 (*c* 1.01, CH_2Cl_2). ^1H NMR (400 MHz, $CDCl_3$) δ 8.29 (d, *J* = 8.6 Hz, 2H), 8.14 – 8.09 (m, 1H), 7.97 – 7.92 (m, 1H), 7.62 (d, *J* = 8.6

Hz, 2H), 7.56 – 7.51 (m, 1H), 7.46 – 7.41 (m, 1H), 7.36 – 7.29 (m, 4H), 7.27 – 7.22 (m, 1H), 4.57 – 4.44 (m, 2H), 4.29 – 4.20 (m, 1H), 3.81 (s, 2H), 2.90 (dd, J = 13.2, 4.7 Hz, 1H), 2.65 (s, 3H), 2.65 – 2.56 (m, 1H). ^{13}C NMR (100 MHz, $CDCl_3$) δ 165.8, 158.5, 154.0, 138.2, 137.3, 136.2, 135.2, 135.0, 134.8, 128.9, 128.6, 128.5, 127.1, 126.6, 125.7, 125.5, 123.5, 121.7, 71.7, 66.6, 36.7, 36.2, 10.2. HRMS-ESI calculated for $[C_{27}H_{23}N_5SO+H]^+$: 498.1417, found 498.1413.

3 NMR spectra

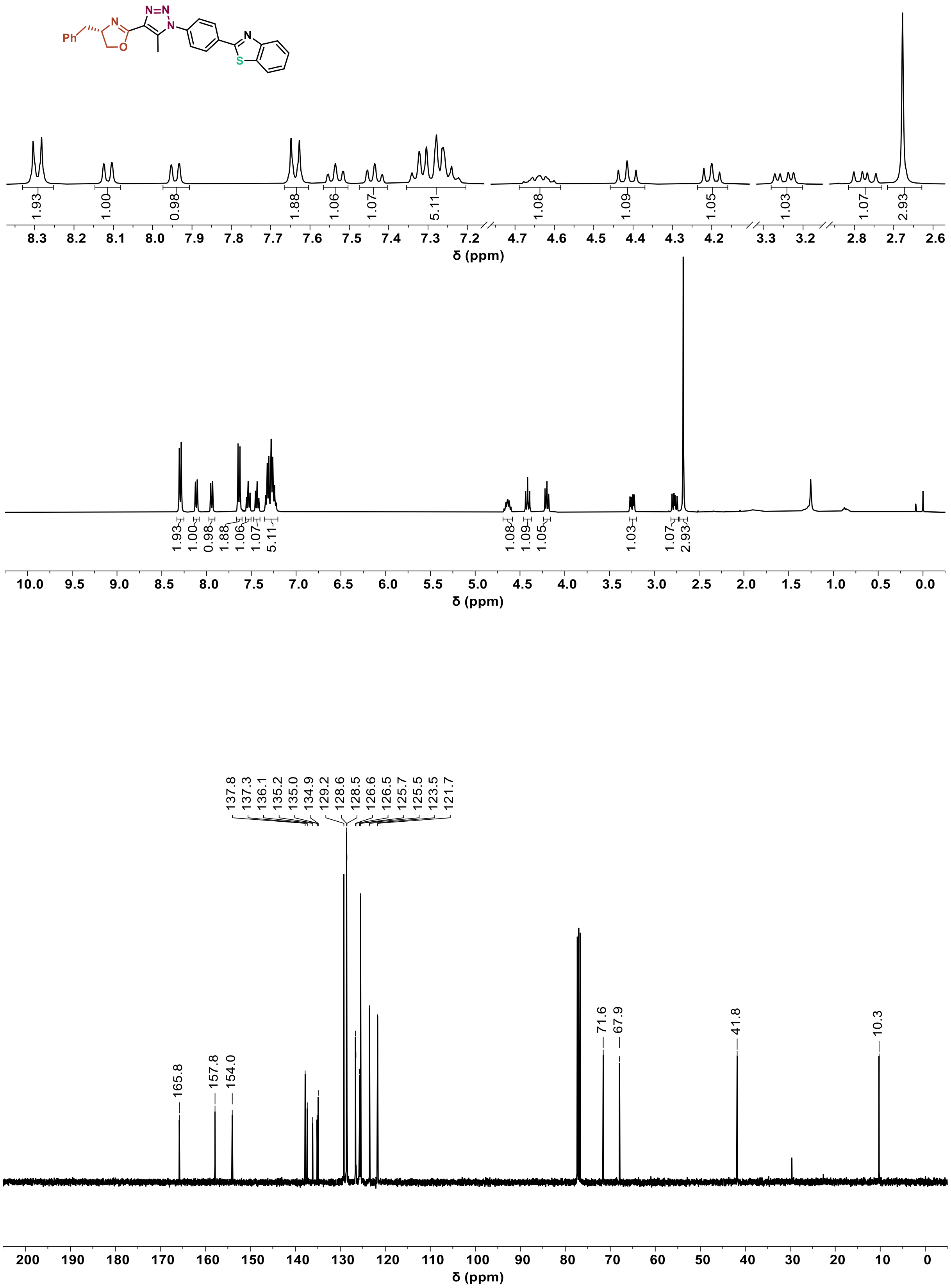

Figure S1. ^{1}H-NMR and ^{13}C-NMR spectra ($CDCl_3$, 400 MHz) for compound **7a**.

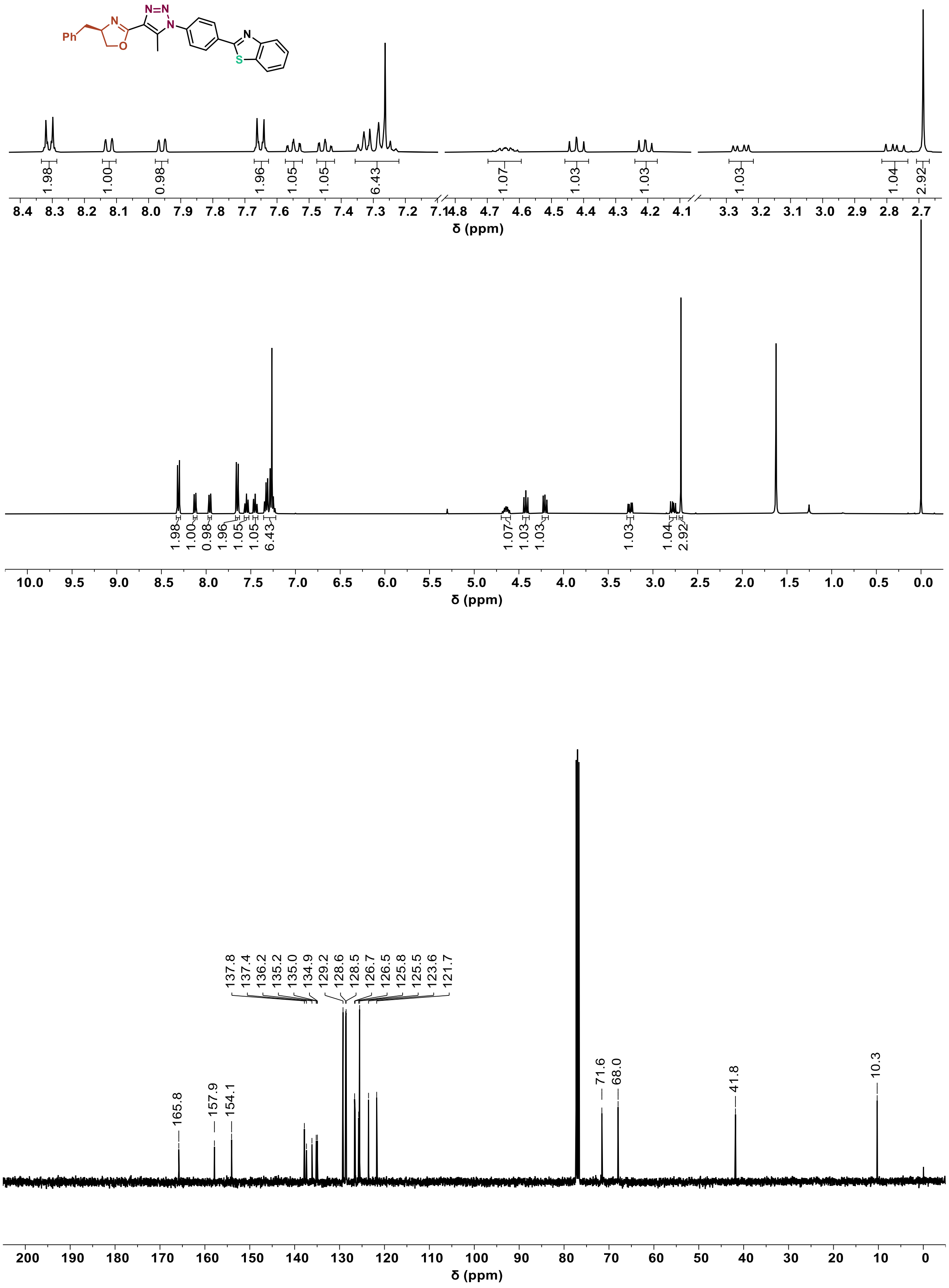

Figure S2. ^{1}H-NMR and ^{13}C-NMR spectra ($CDCl_3$, 400 MHz) for compound **7b**.

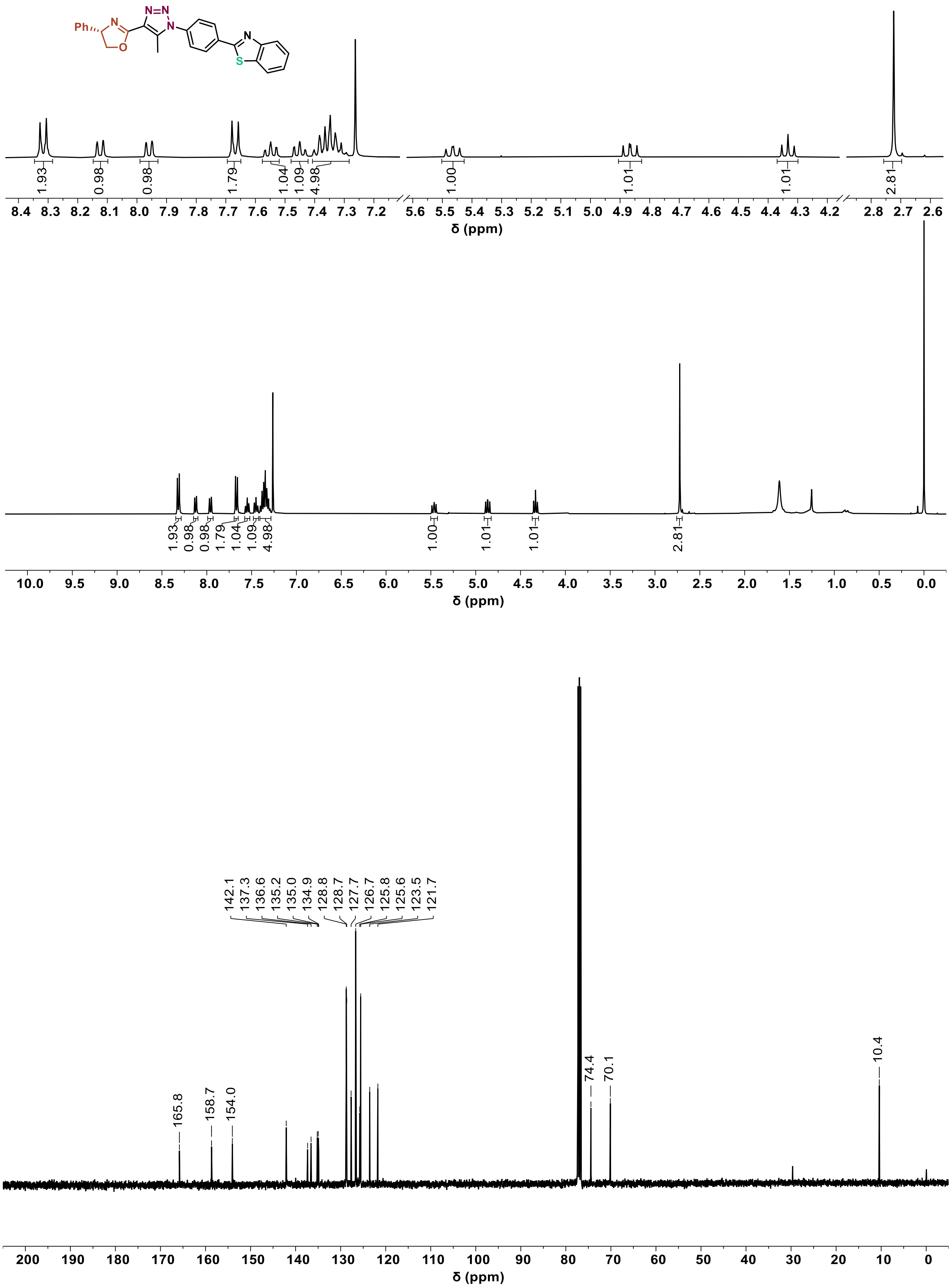

Figure S3. ^{1}H-NMR and ^{13}C-NMR spectra ($CDCl_3$, 400 MHz) for compound **7c**.

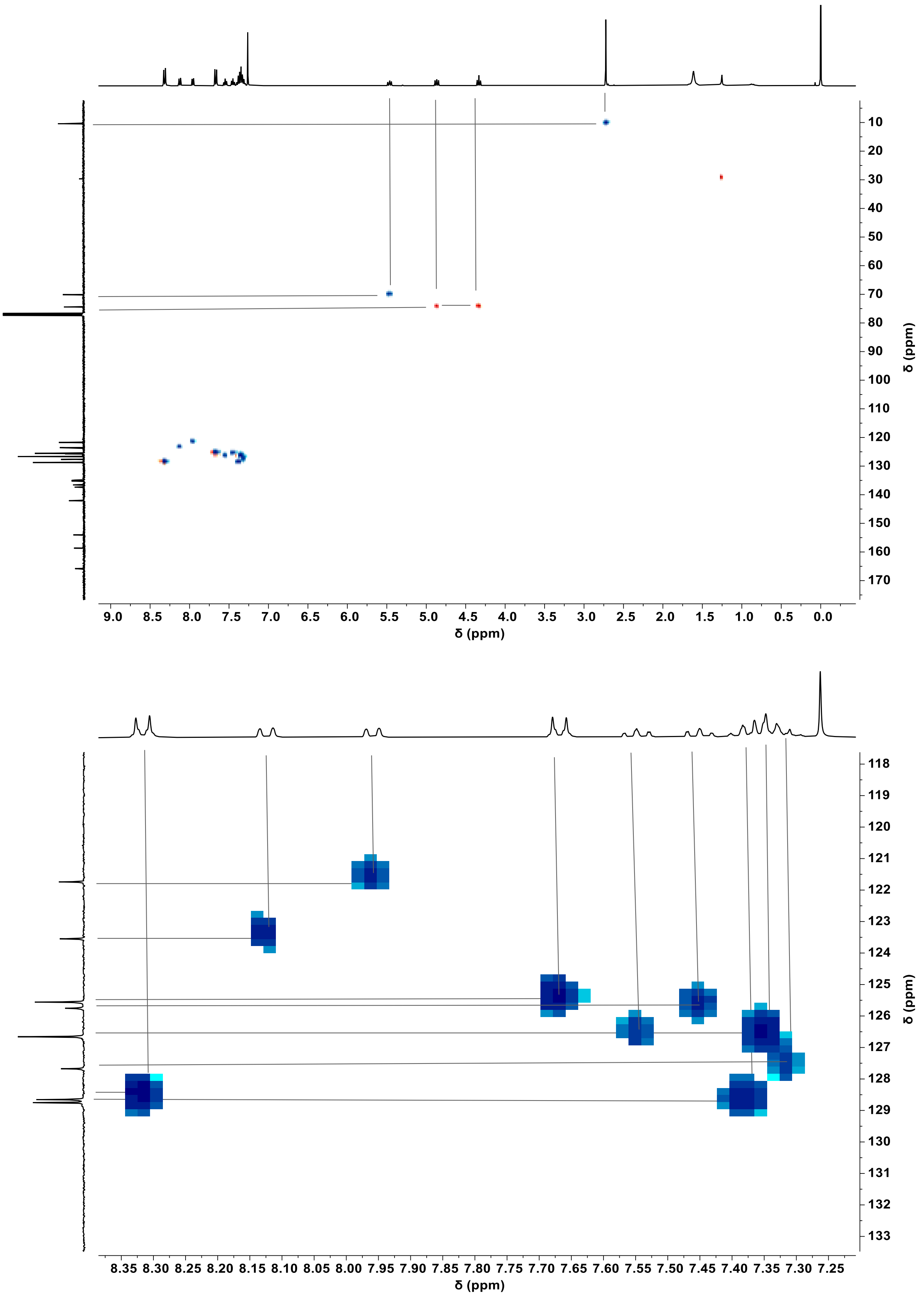

Figure S4. ^{1}H-^{13}C-HSQC NMR spectrum ($CDCl_3$, 400 MHz) for compound **7c**.

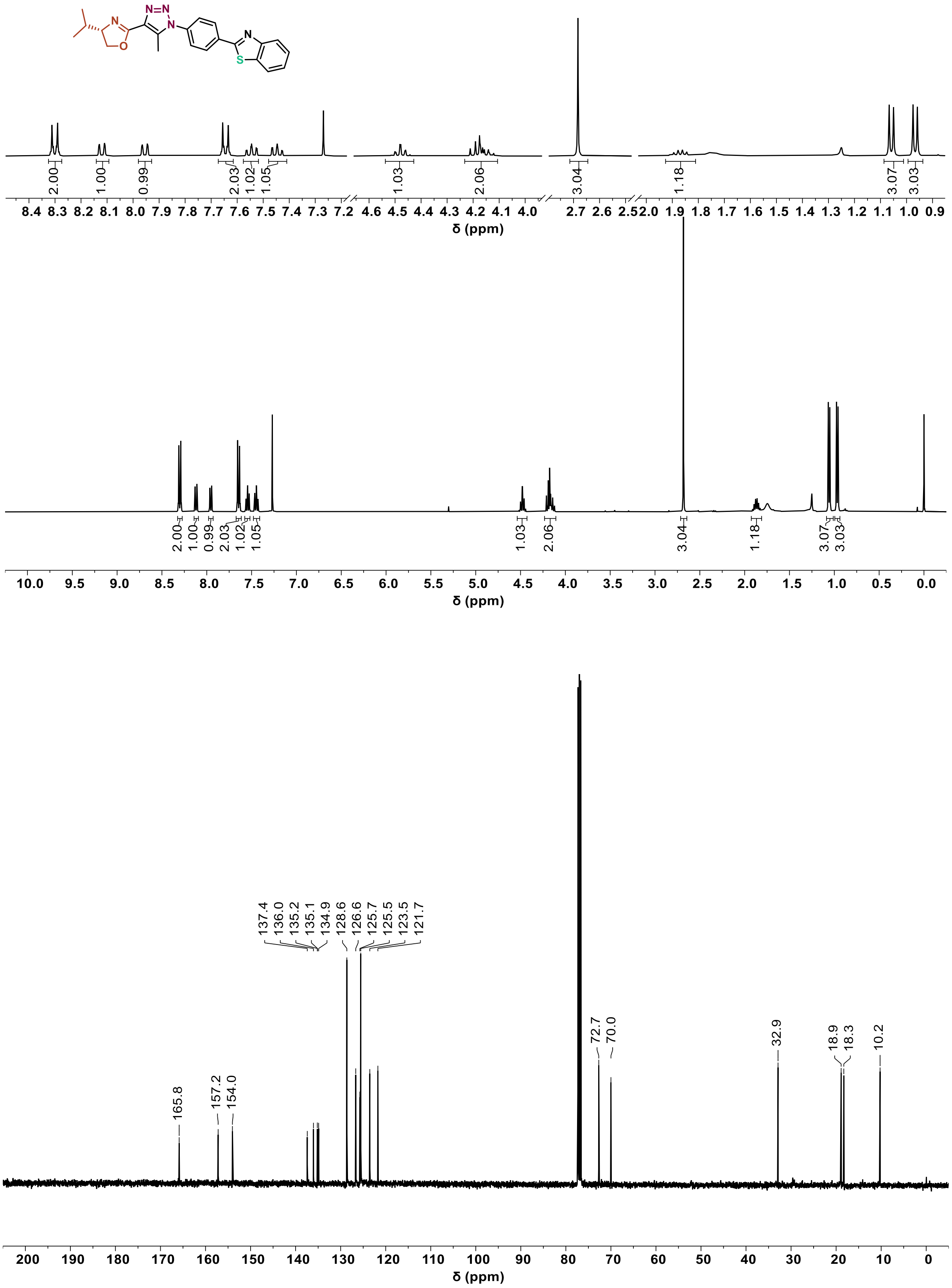

Figure S5. ^{1}H-NMR and ^{13}C-NMR spectra ($CDCl_3$, 400 MHz) for compound **7d**.

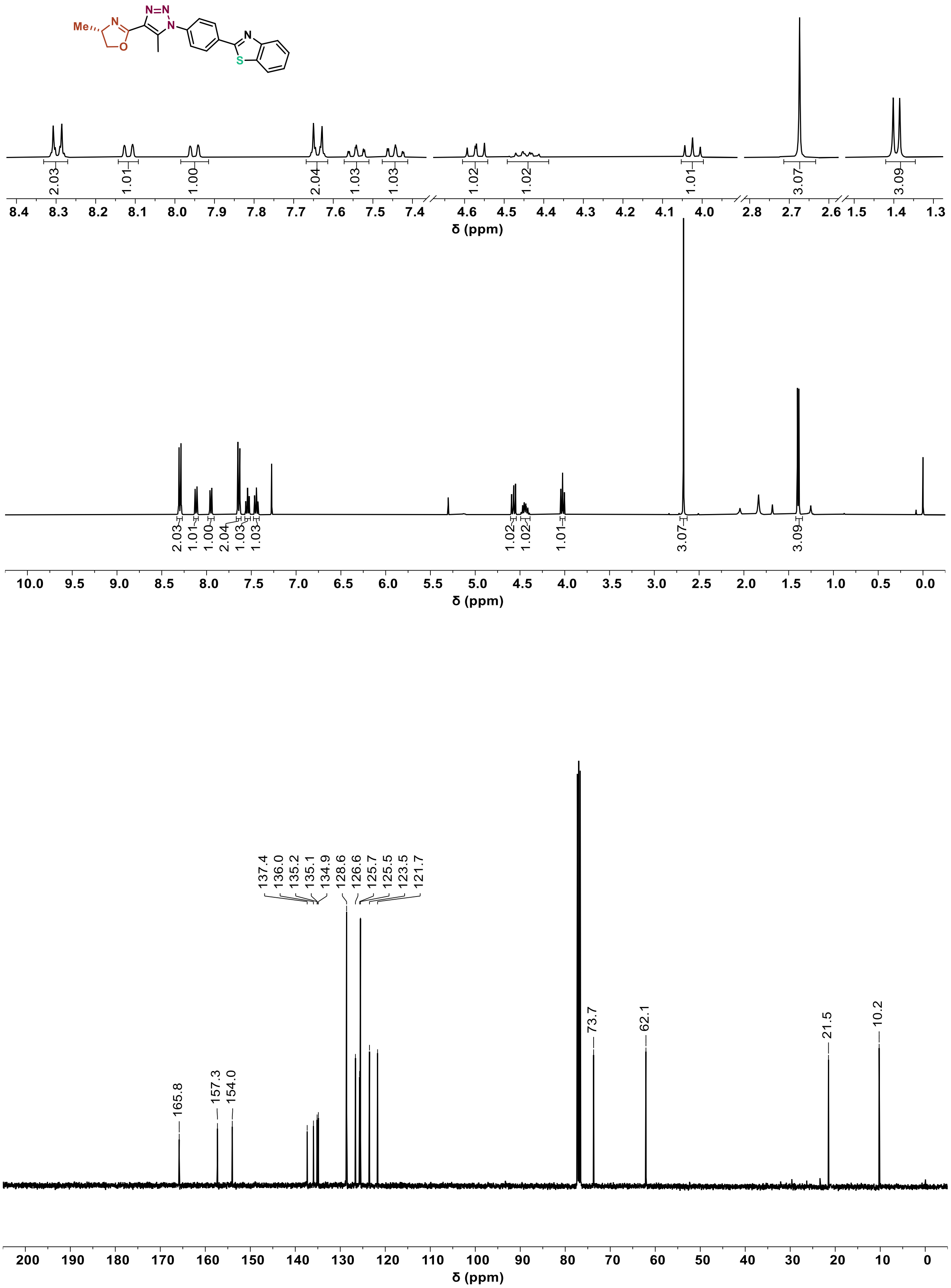

Figure S6. ^{1}H-NMR and ^{13}C-NMR spectra ($CDCl_3$, 400 MHz) for compound **7e**.

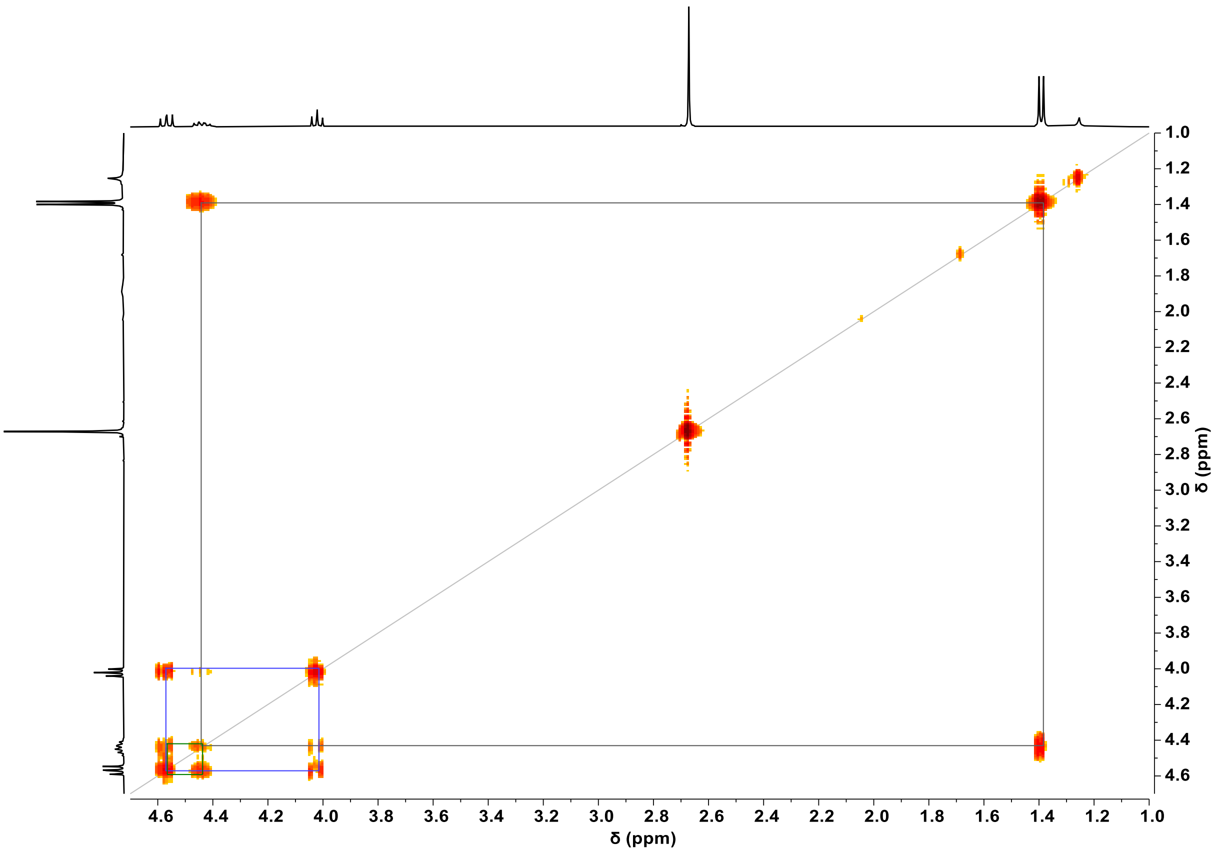

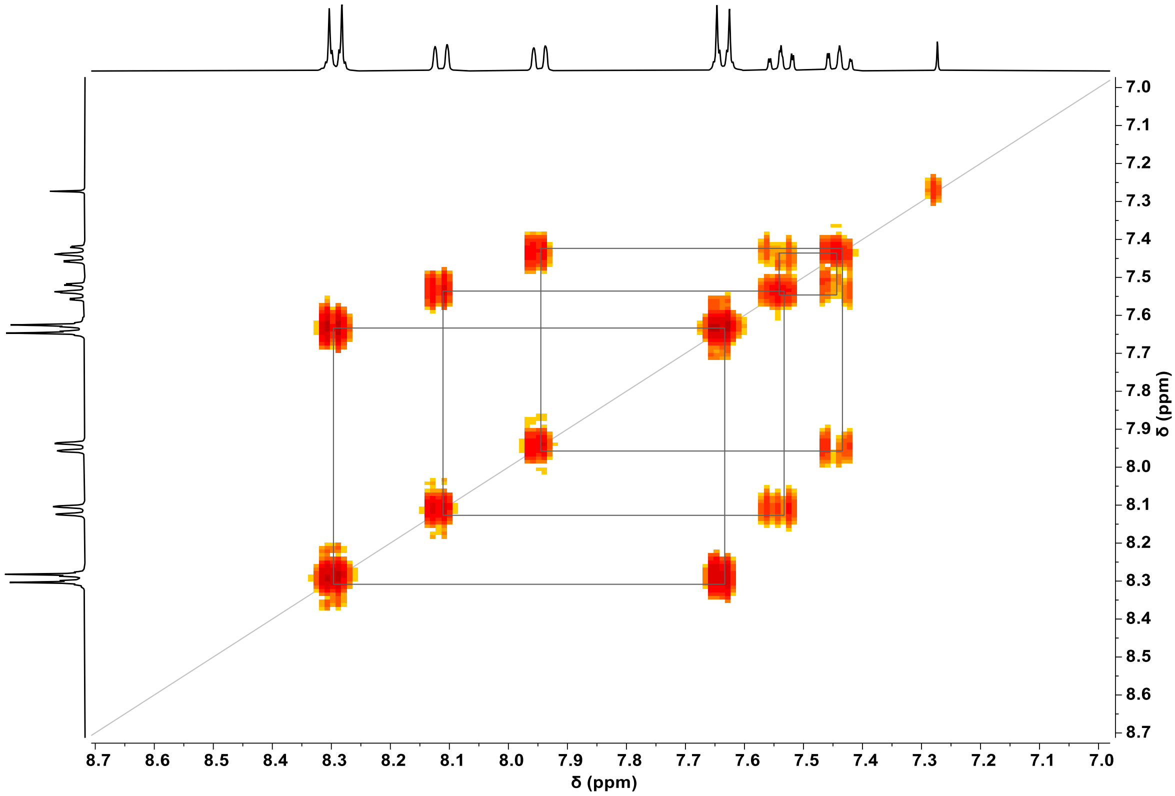

Figure S7. Aliphatic (top) and aromatic (bottom) regions ampliations of ^{1}H-^{1}H-COSY NMR spectrum ($CDCl_3$, 400 MHz) for compound **7e**.

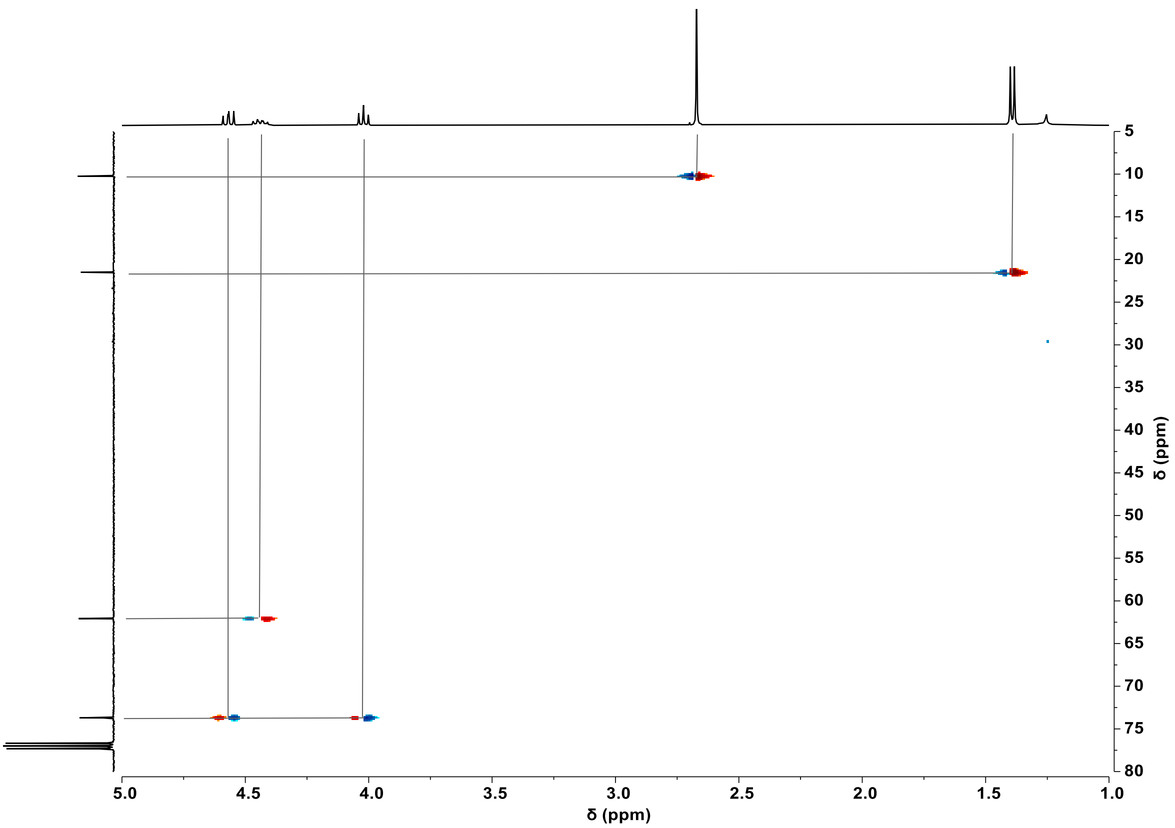

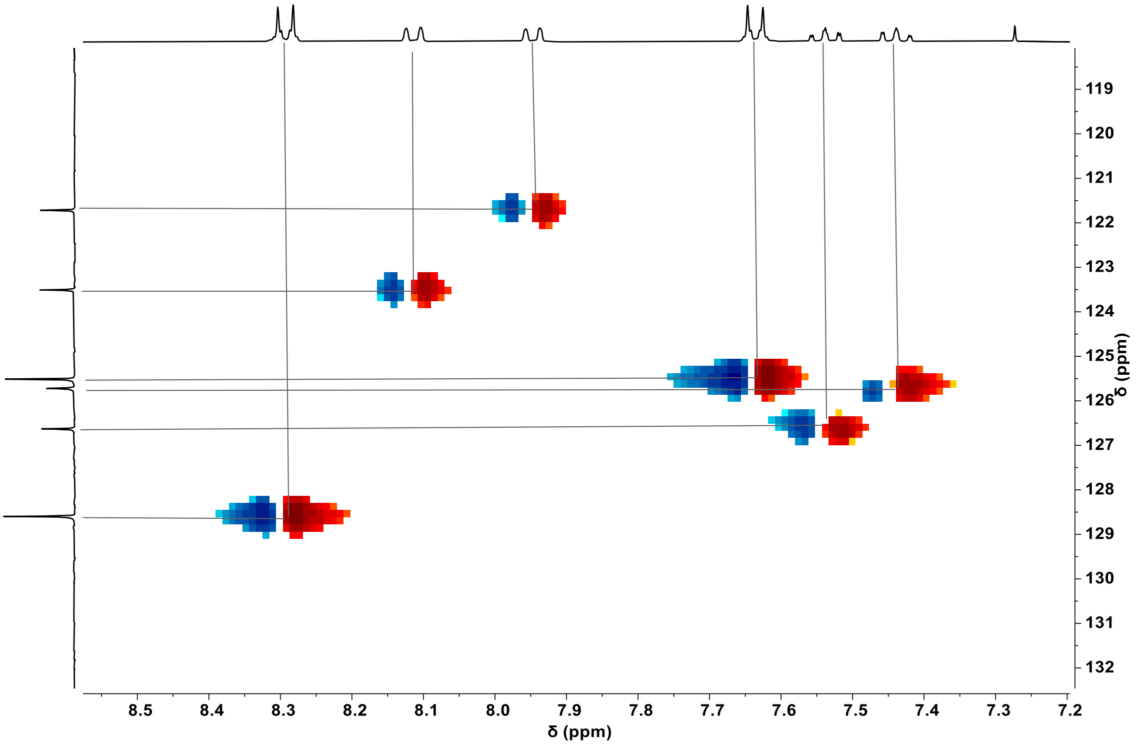

Figure S8. Aliphatic (top) and aromatic (bottom) regions ampliations of ^{1}H-^{13}C-HSQC NMR spectrum ($CDCl_3$, 400 MHz) for compound **7e.**

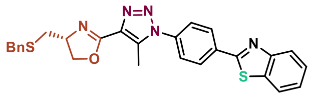

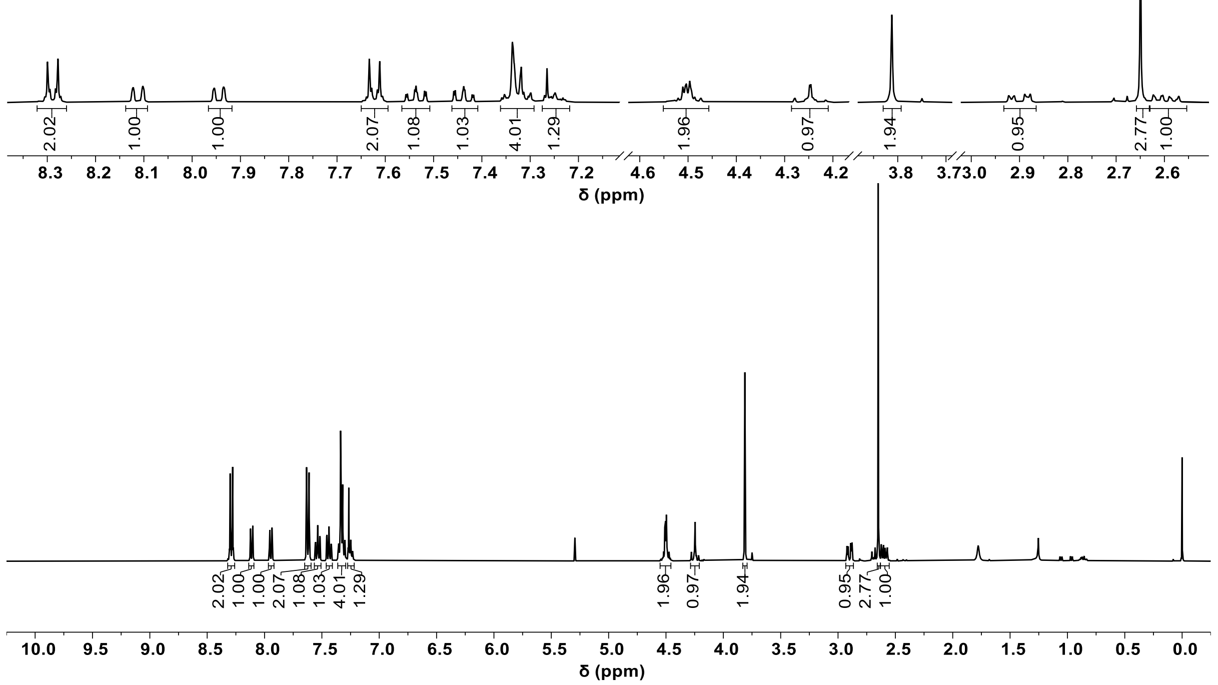

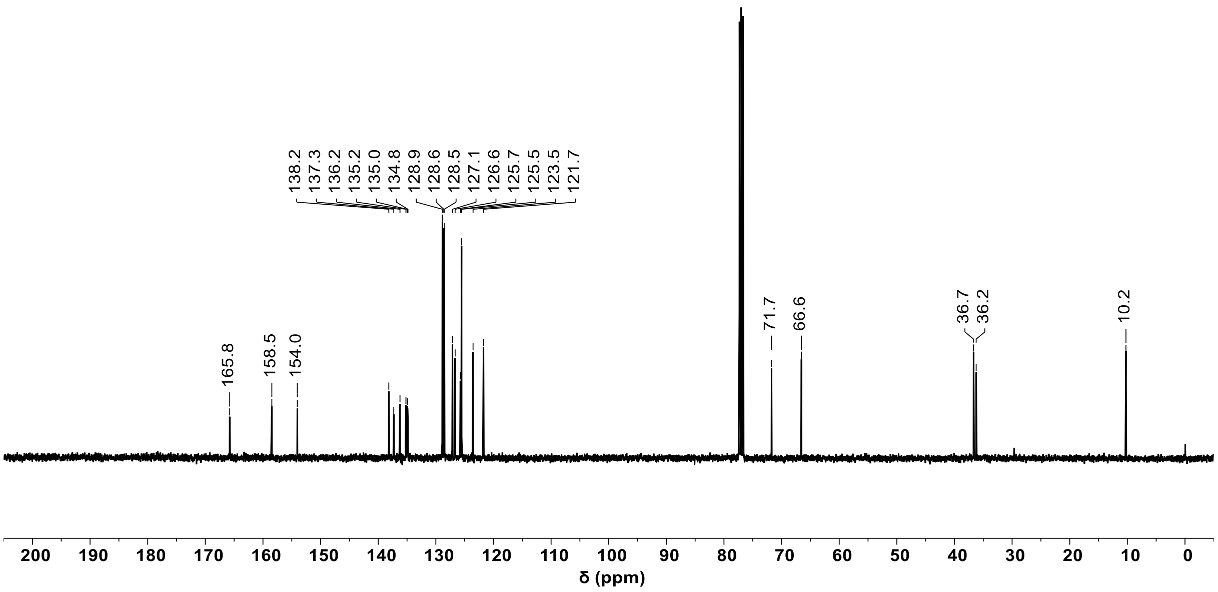

Figure S9. ^{1}H-NMR and ^{13}C-NMR spectra ($CDCl_3$, 400 MHz) for compound **7f**.

4 Absorption, emission and excitation spectra

Table S1. Working concentrations

Sample	Final concentrations (Molar)
**7a**	1.03E-05
**7b**	1.11E-05
**7c**	9.52E-06
**7d**	1.24E-05
**7e**	1.21E-05
**7f**	8.37E-06

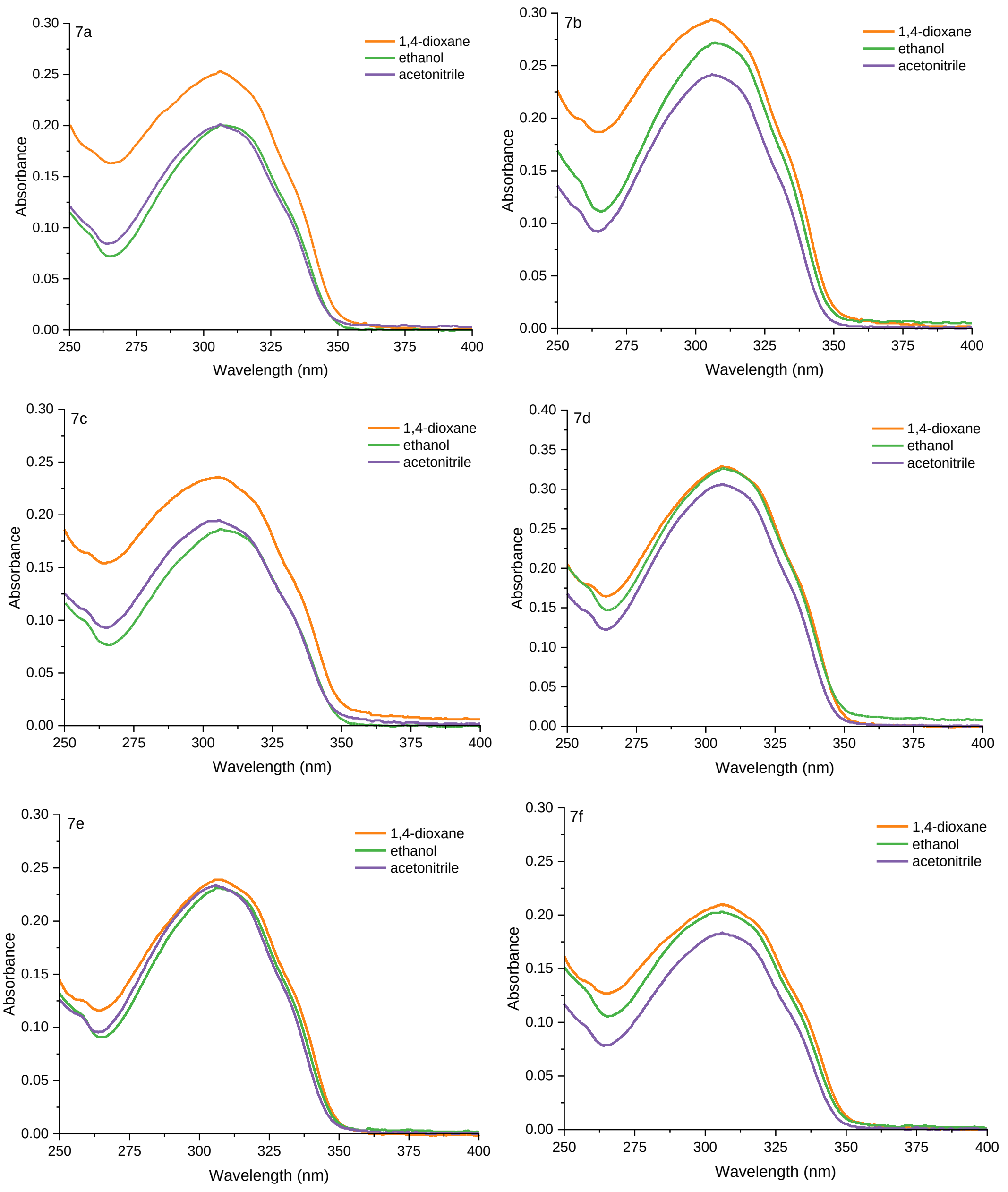

Figure S10. UV-Vis absorption spectra of dyes **7a-f**.in solution of different organic solvents (ca 10^{-5} M).

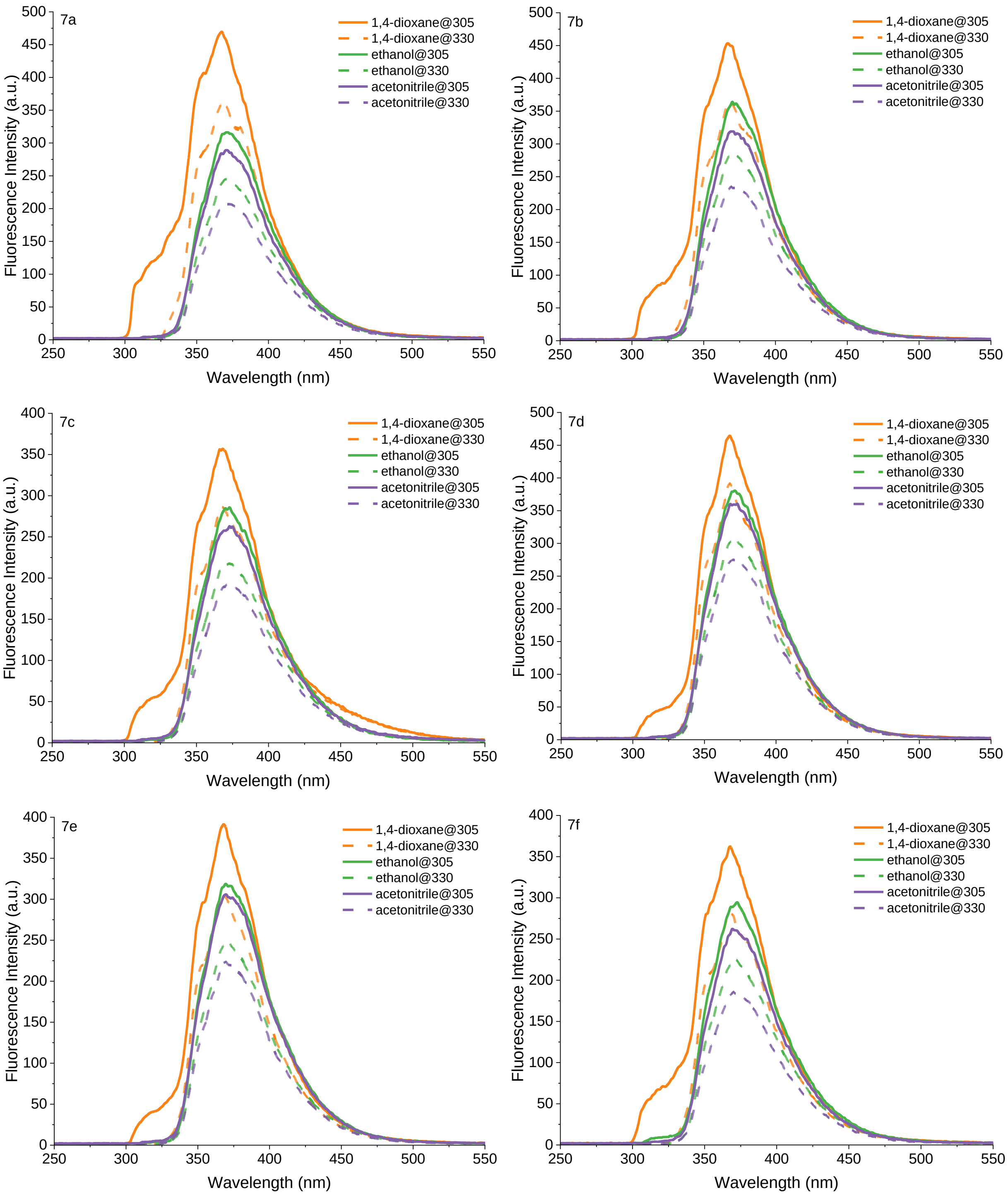

Figure S11. Steady-state fluorescence emission spectra of dyes **7a-f** in solution of different organic solvents (ca 10^{-5} M) at λ_{exc} 305 e 330 nm (exc./em. slits 3.0/3.0 nm).

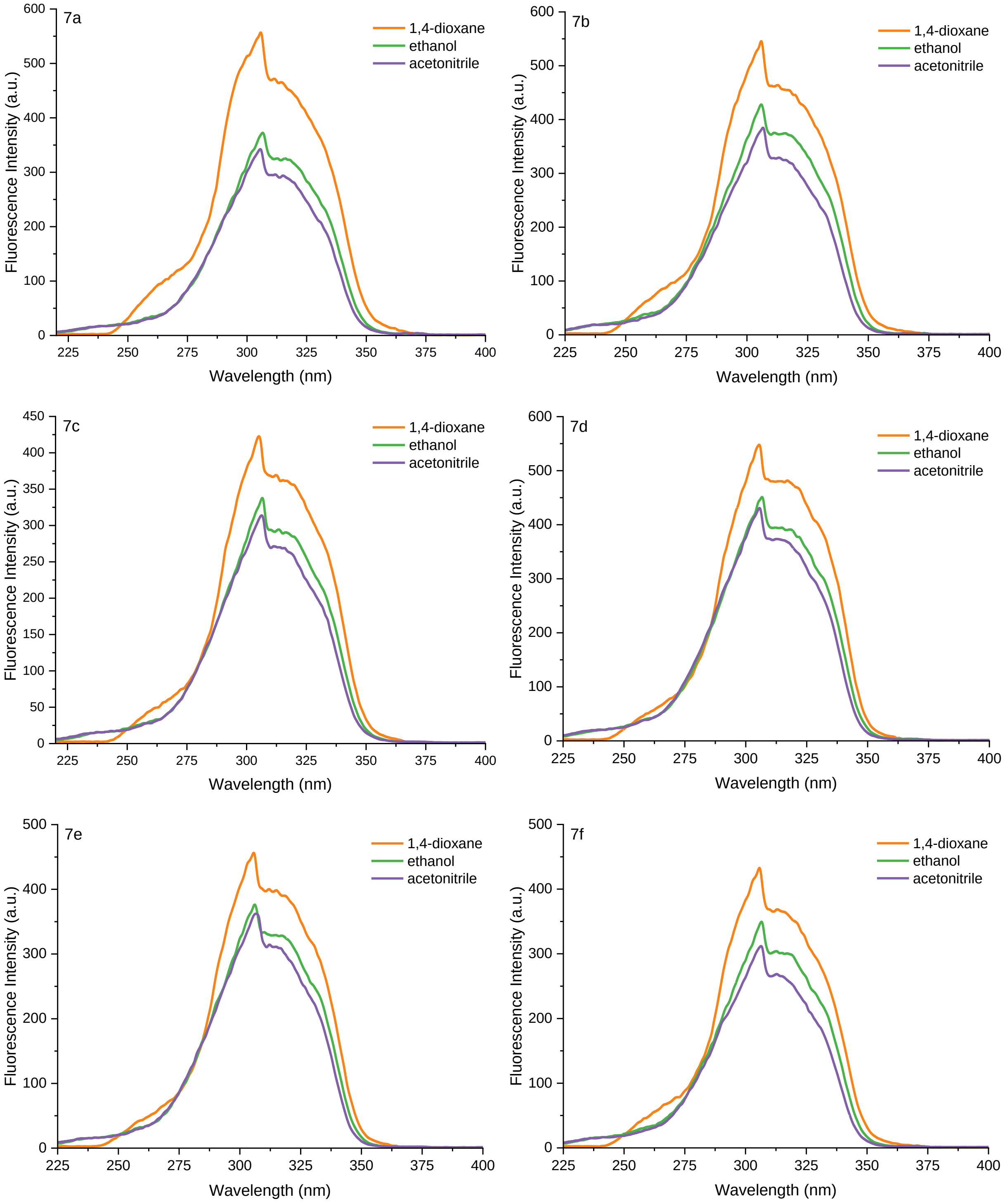

Figure S12 Excitation spectra of dyes **7a-f** in solution of different organic solvents (ca 10^{-5} M, $\lambda_{em.}$ = 370 nm, exc./em. slits 3.0/3.0 nm).

5 Additional data for arabinose interaction study

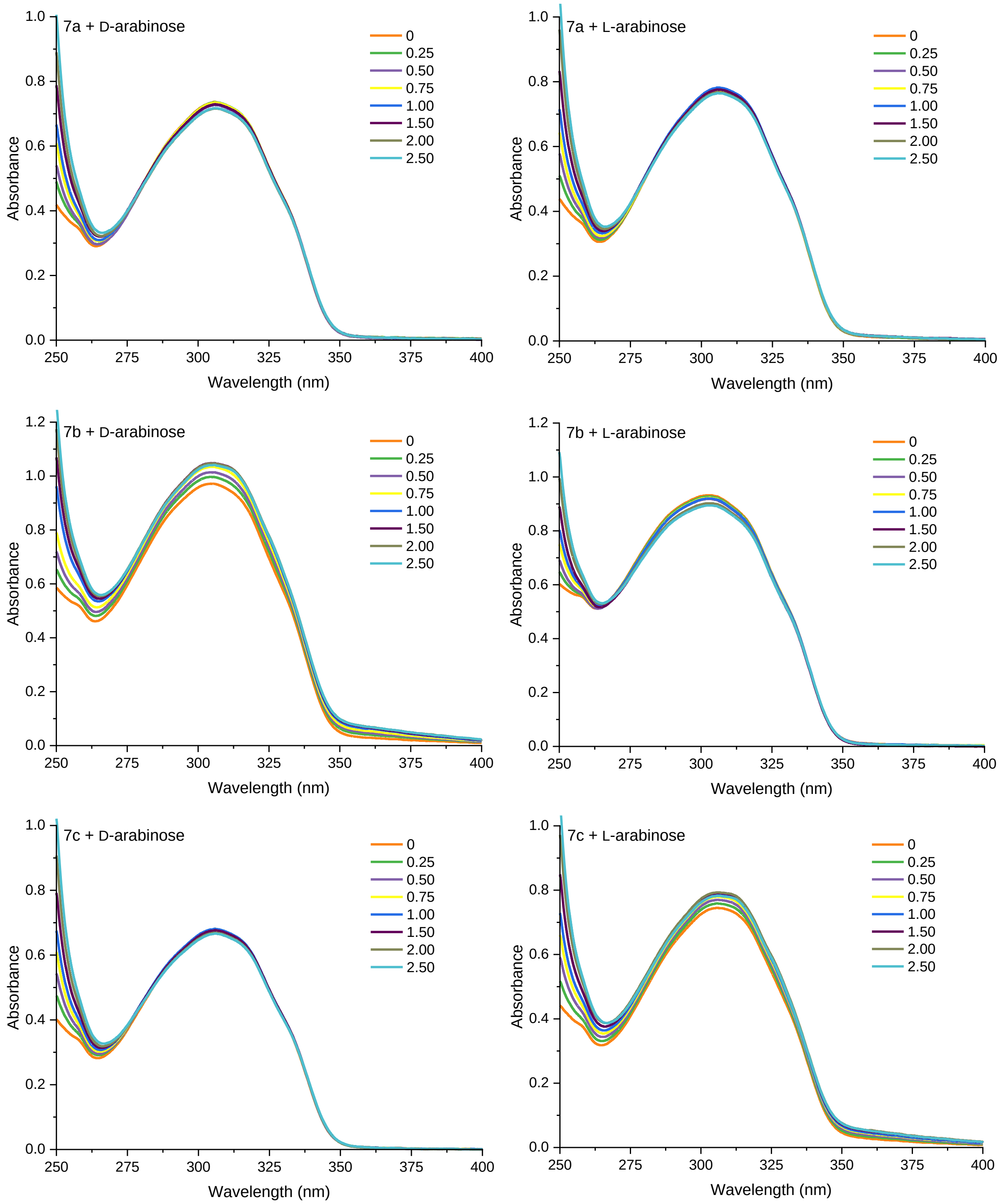

Figure S13. UV-Vis spectra of dyes **7a-c** in acetonitrile (**7a**: 4.13x10^{-5} M, **7b**: 4.43x10^{-5} M, and **7c**: 3.81x10^{-5} M) with different amounts (0-2.50 equiv.) of D- and L-arabinose in DMSO solution.

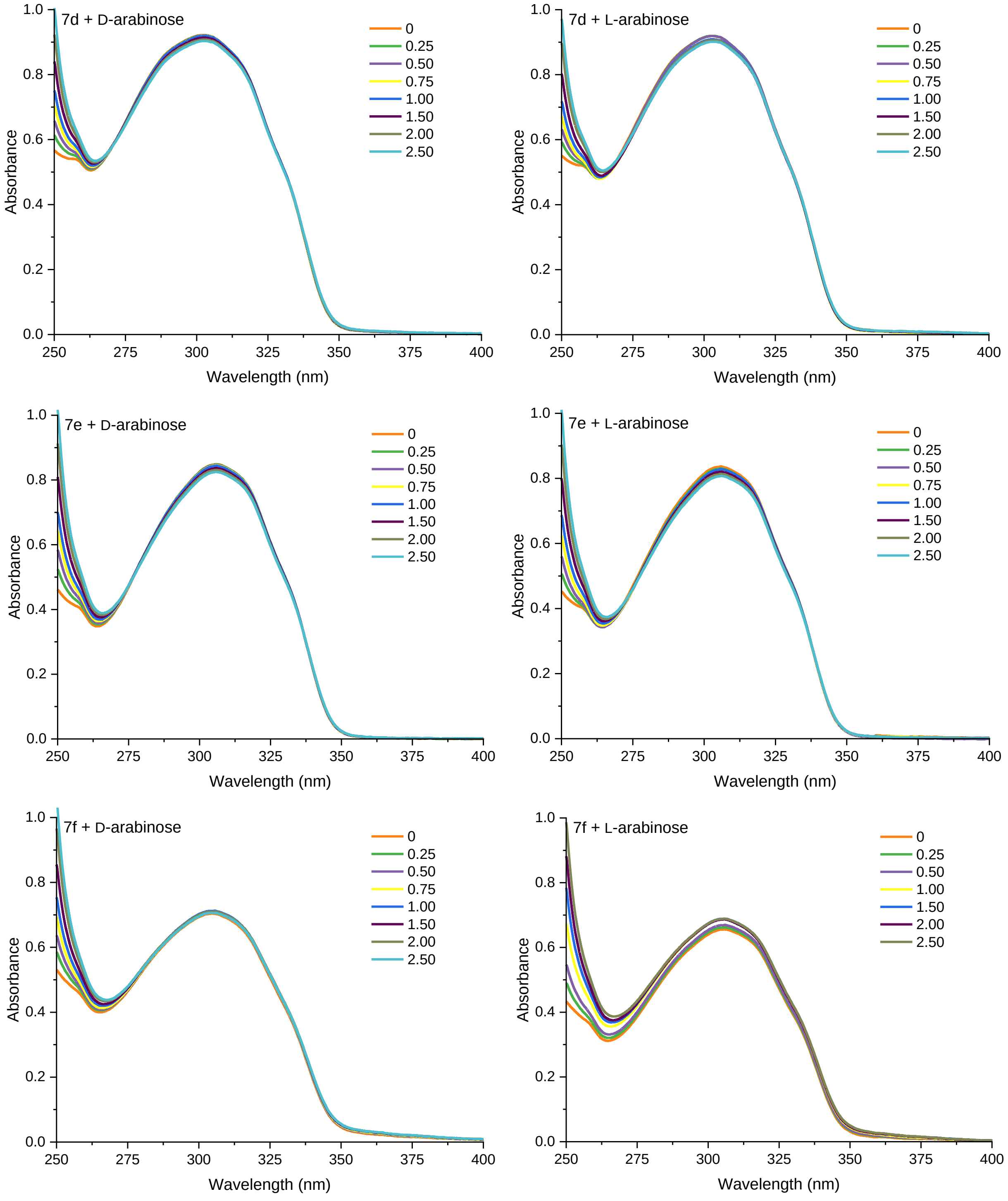

Figure S14. UV-Vis spectra of dyes **7d-f** in acetonitrile (**7d**: 4.13x10^{-5} M, **7e**: 4.85x10^{-5} M, and **7f**: 3.35x10^{-5} M) with different amounts (0-2.50 equiv.) of D- and L-arabinose in DMSO solution.

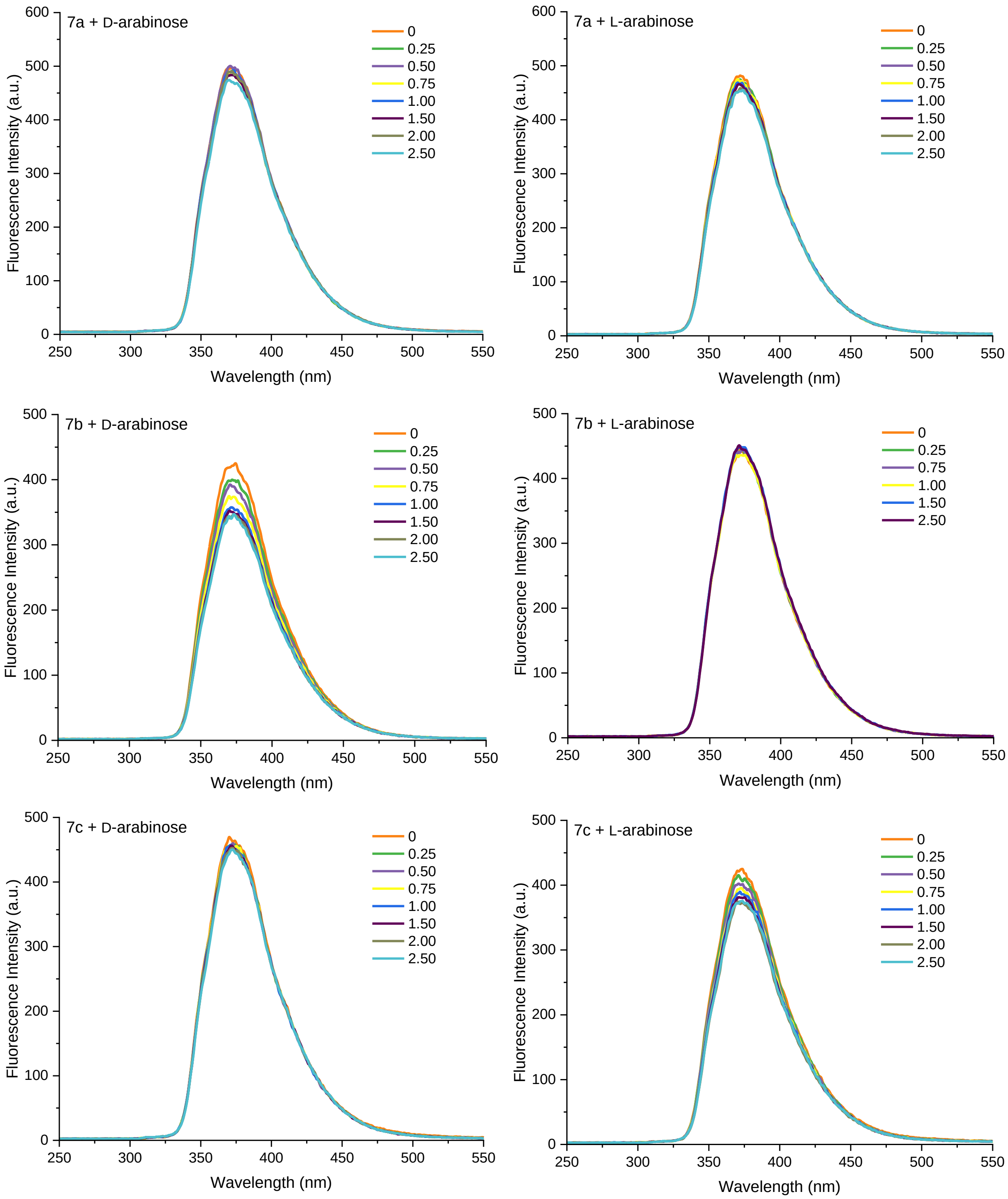

Figure S15. Fluorescence spectra of dyes **7a-c** (**7a**: 4.13x10^{-5} M, **7b**: 4.43x10^{-5} M, and **7c**: 3.81x10^{-5} M) in acetonitrile with different amounts (0-2.50 equiv.) of D- and L-arabinose in DMSO solution. (λ_{exc} = 305 nm, exc./em. slits 3.0/3.0 nm)

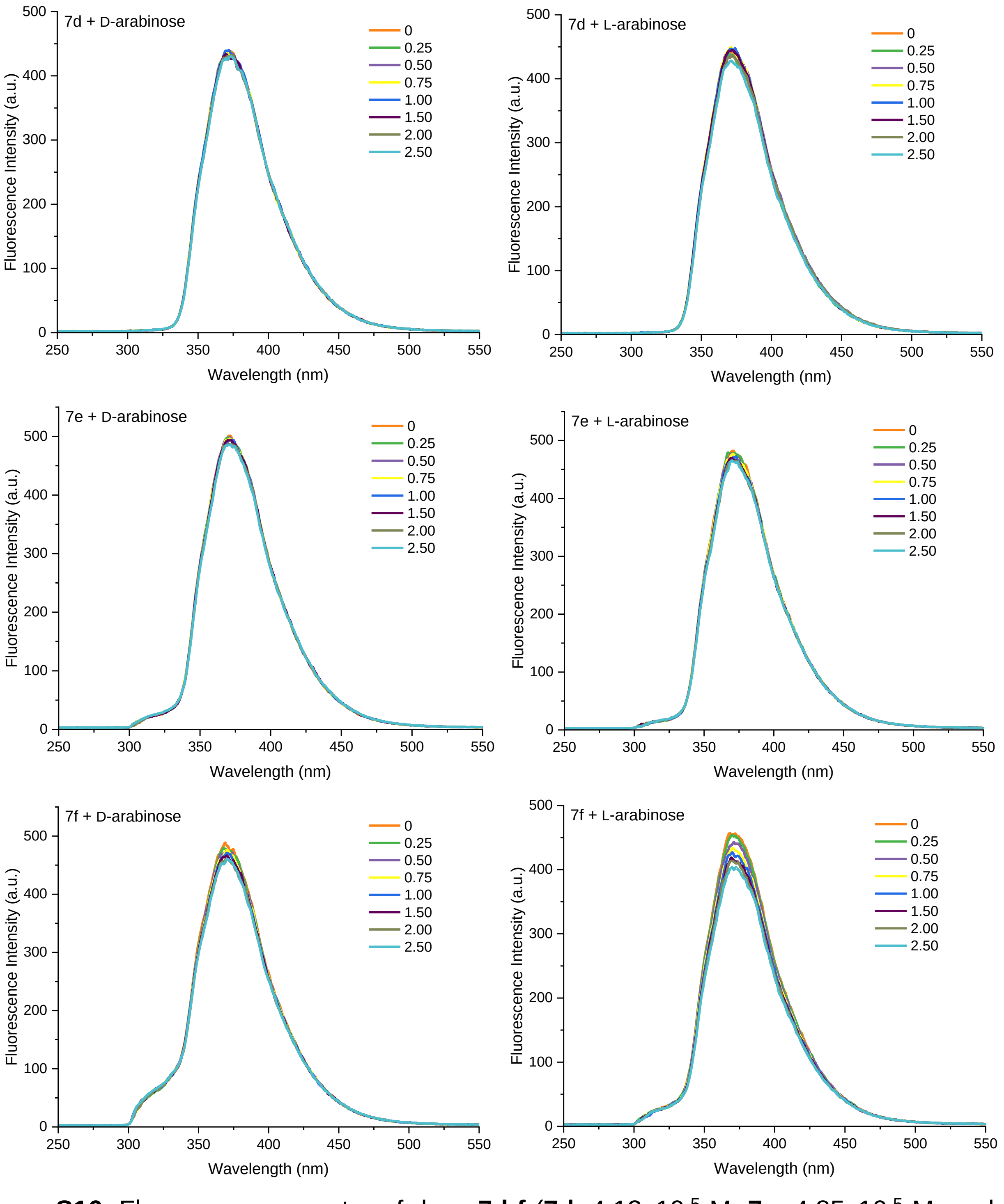

Figure S16. Fluorescence spectra of dyes **7d-f** (**7d**: 4.13x10^{-5} M, **7e**: 4.85x10^{-5} M, and **7f**: 3.35x10^{-5} M) in acetonitrile with different amounts (0-2.50 equiv.) of D- and L-arabinose in DMSO solution. (λ_{exc} = 305 nm, exc./em. slits 3.0/3.0 nm)

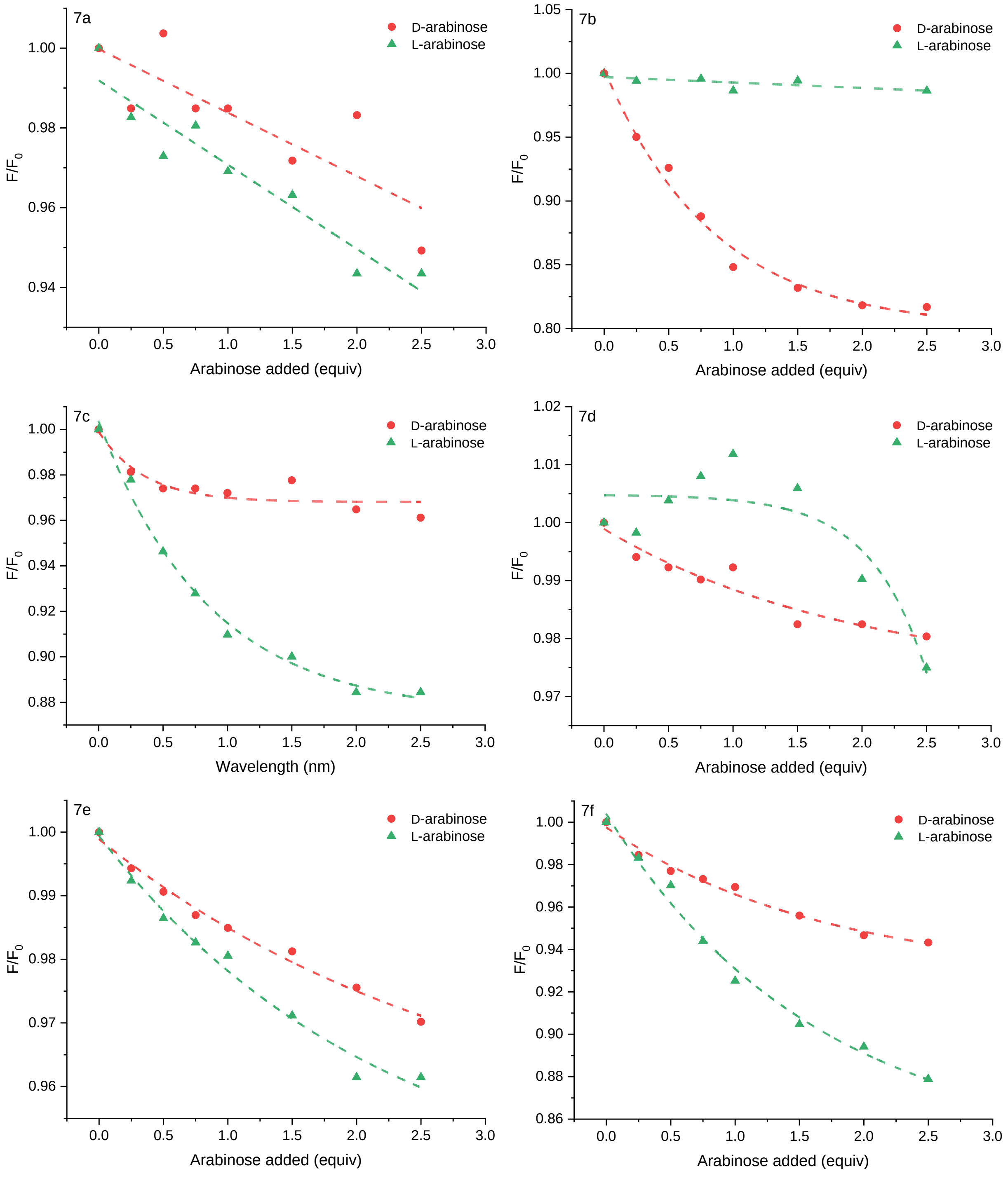

Figure S17. Graphical of the ratio of the fluorescence emission of dyes **7a-f** *versus* different amounts (0-2.50 equiv.) of D- and L-arabinose at 305 nm.

6 Additional data for mannose interaction study

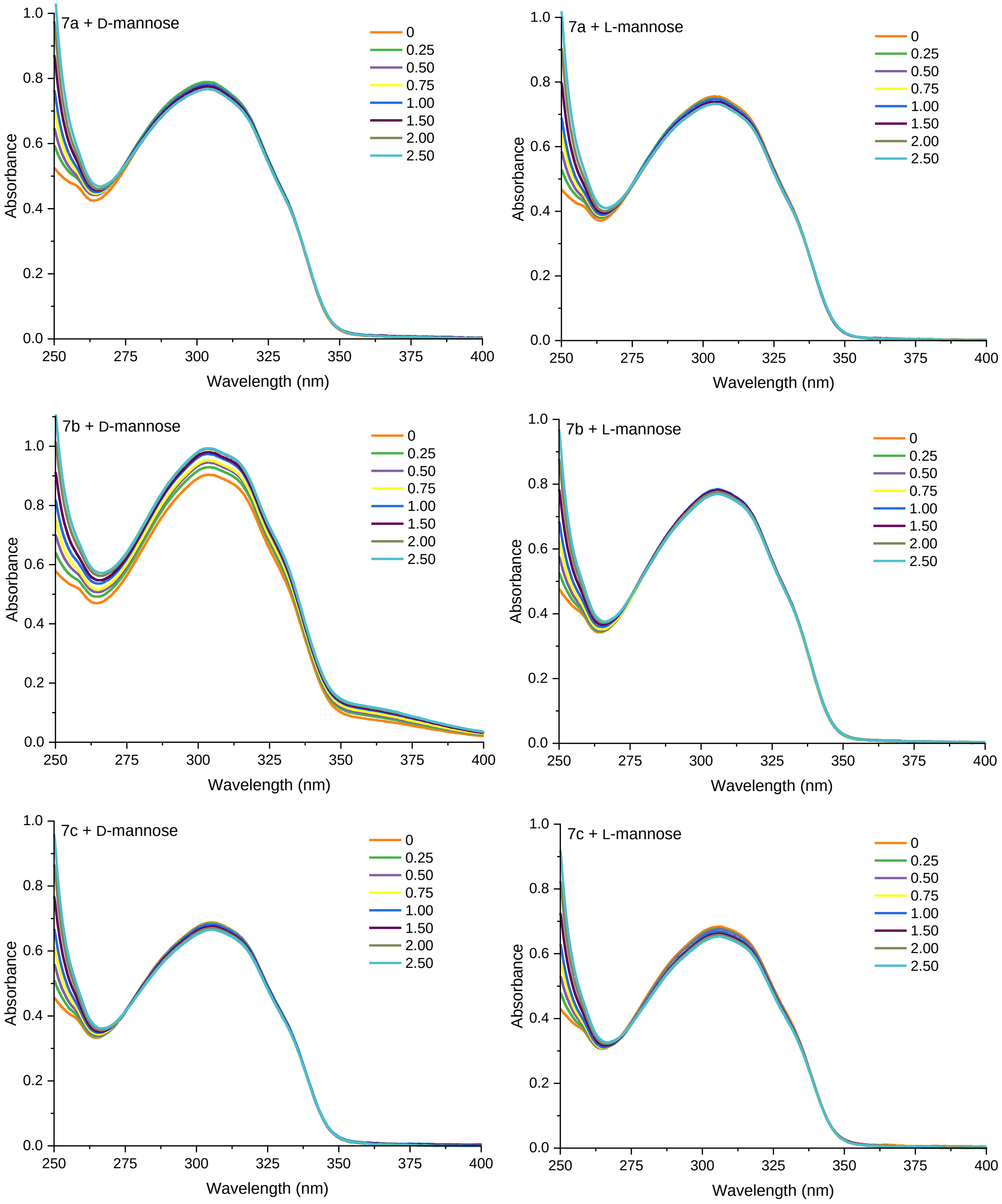

Figure S18. UV-Vis spectra of dyes **7a-c** in acetonitrile (**7a**: 4.13x10^{-5} M, **7b**: 4.43x10^{-5} M, and **7c**: 3.81x10^{-5} M) with different amounts (0-2.50 equiv.) of D- and L-mannose in DMSO solution.

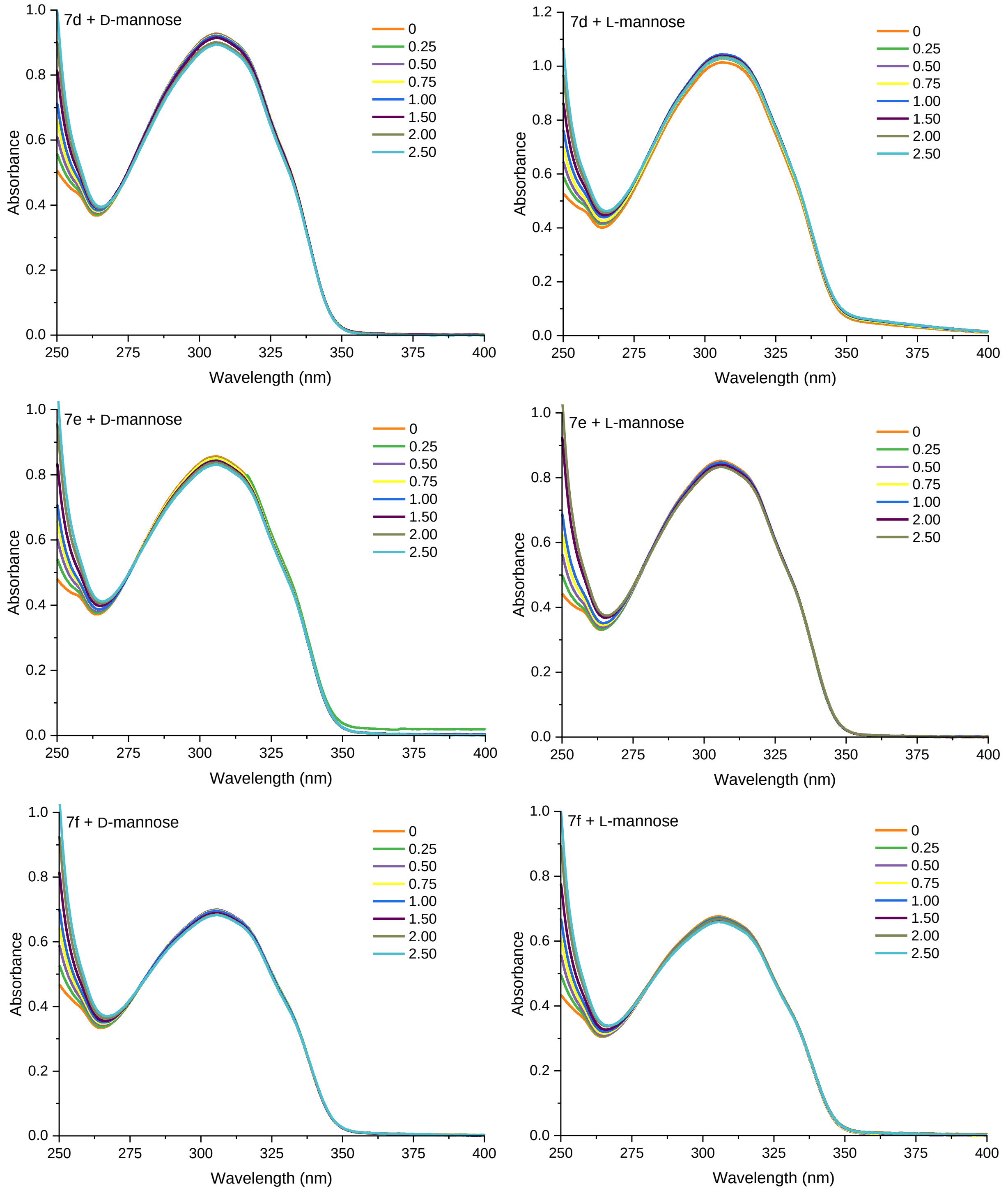

Figure S19. UV-Vis spectra of dyes **7d-f** in acetonitrile (**7d**: 4.13x10^{-5} M, **7e**: 4.85x10^{-5} M, and **7f**: 3.35x10^{-5} M) with different amounts (0-2.50 equiv.) of D- and L-mannose in DMSO solution.

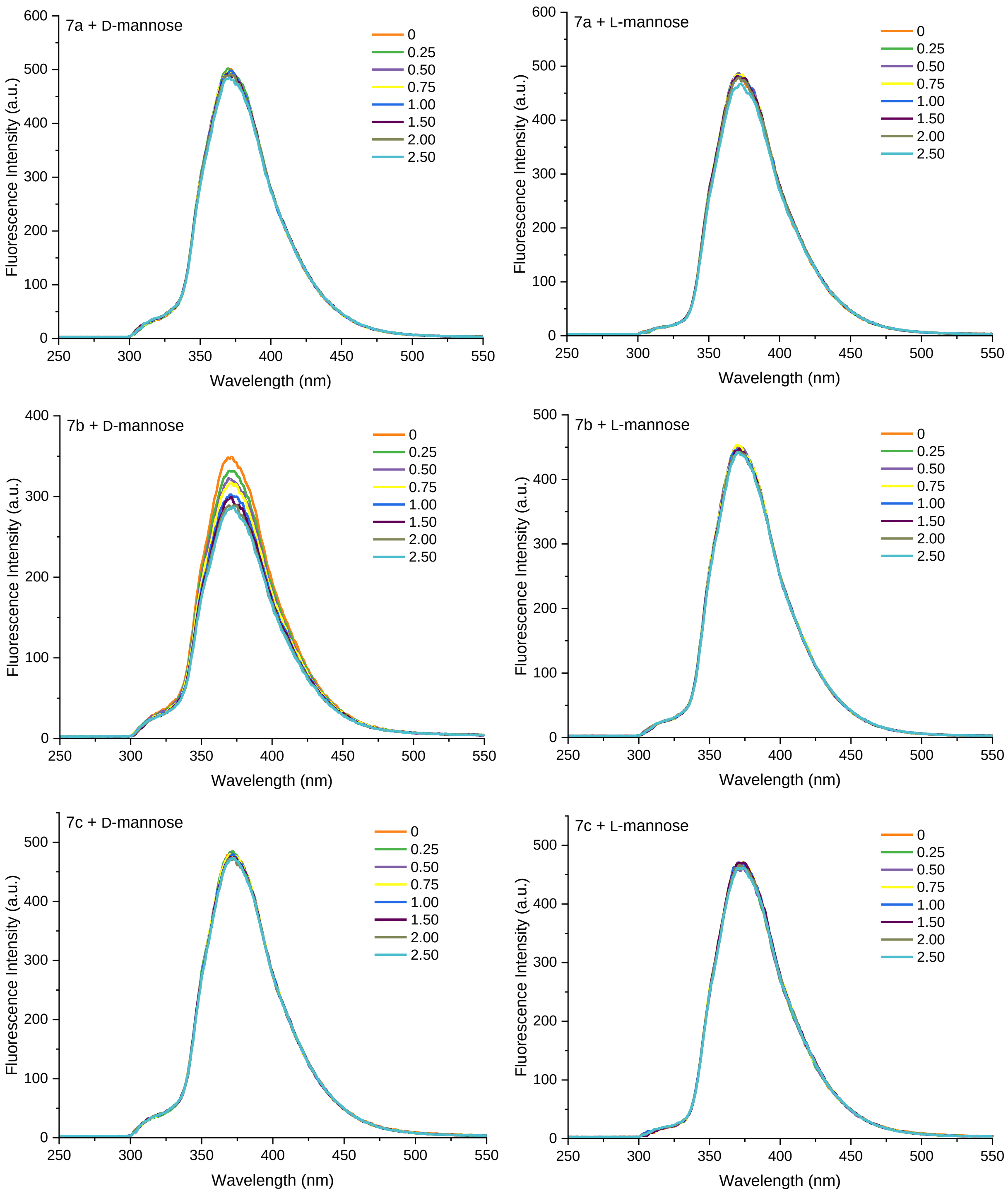

Figure S20. Fluorescence spectra of dyes **7a-c** (**7a**: 4.13x10^{-5} M, **7b**: 4.43x10^{-5} M, and **7c**: 3.81x10^{-5} M) in acetonitrile with different amounts (0-2.50 equiv.) of D- and L-mannose in DMSO solution. (λ_{exc} = 305 nm, exc./em. slits 3.0/3.0 nm)

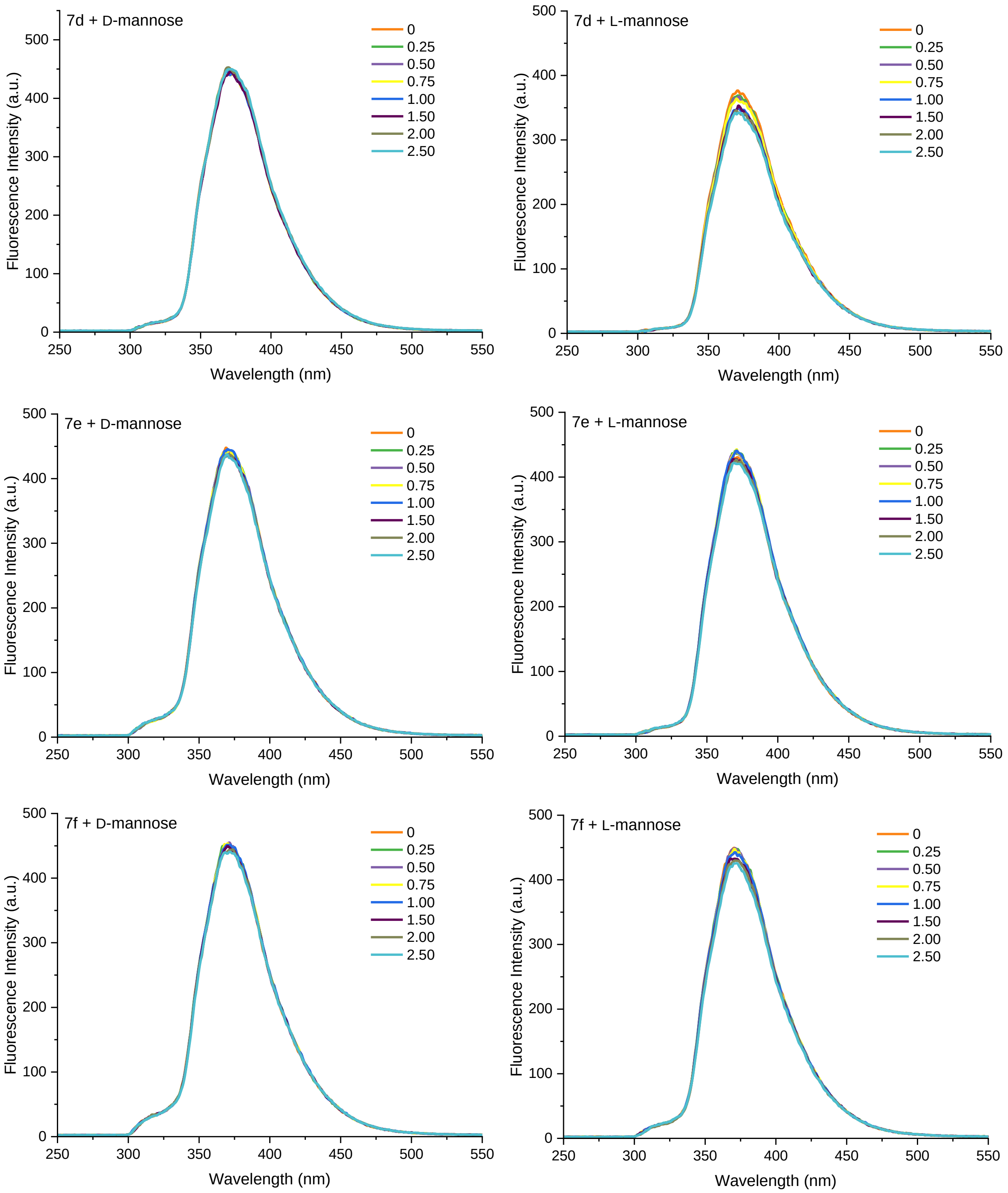

Figure S21. Fluorescence spectra of dyes **7d-f** (**7d**: 4.13x10^{-5} M, **7e**: 4.85x10^{-5} M, and **7f**: 3.35x10^{-5} M) in acetonitrile with different amounts (0-2.50 equiv.) of D- and L-mannose in DMSO solution. (λ_{exc} = 305 nm, exc./em. slits 3.0/3.0 nm)

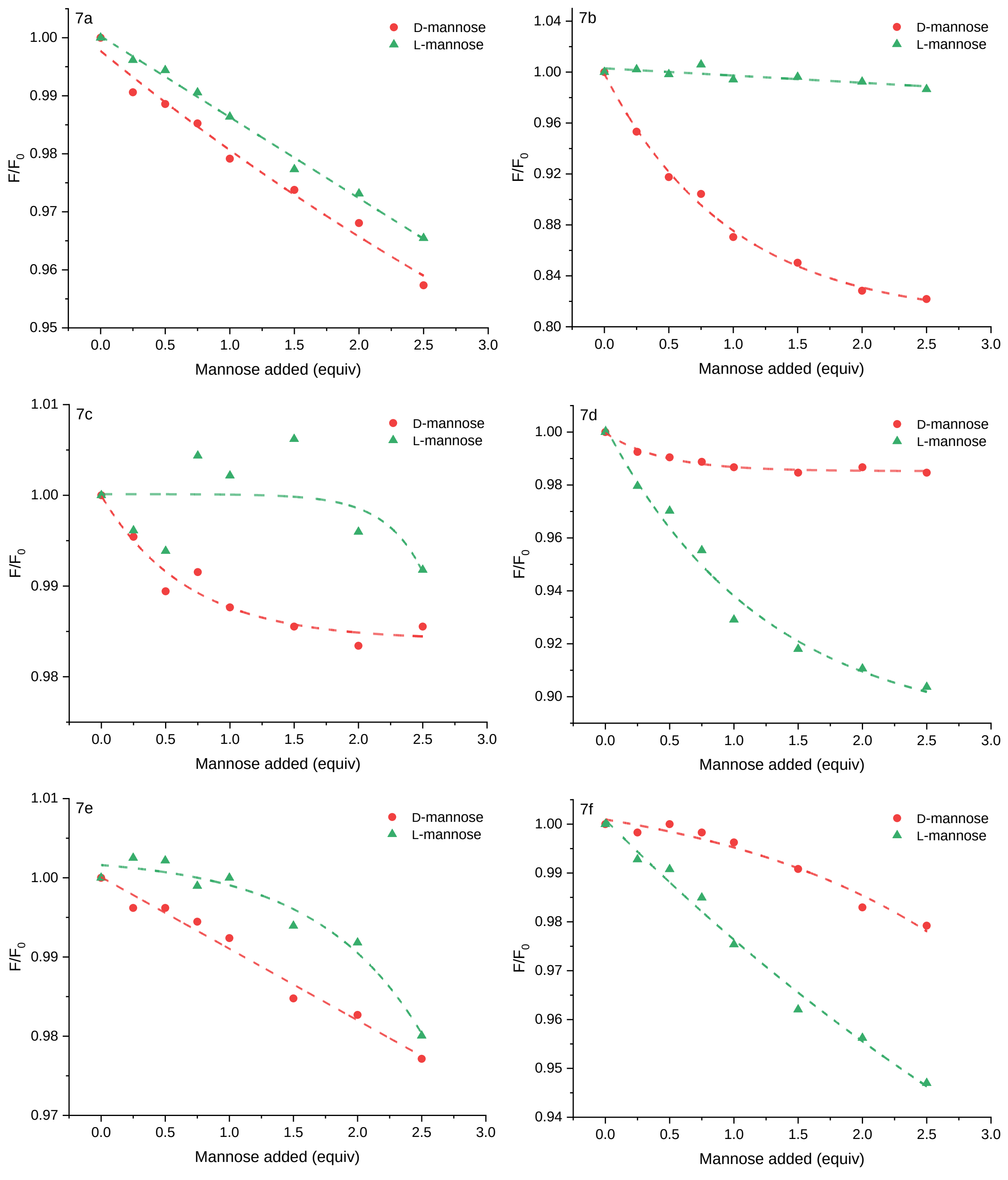

Figure S22. Graphical of the ratio of the fluorescence emission of dyes **7a-f** *versus* different amounts (0-2.50 equiv.) of D- and L-mannose at 305 nm.

7 Additional data for glucose and xylose interaction studies

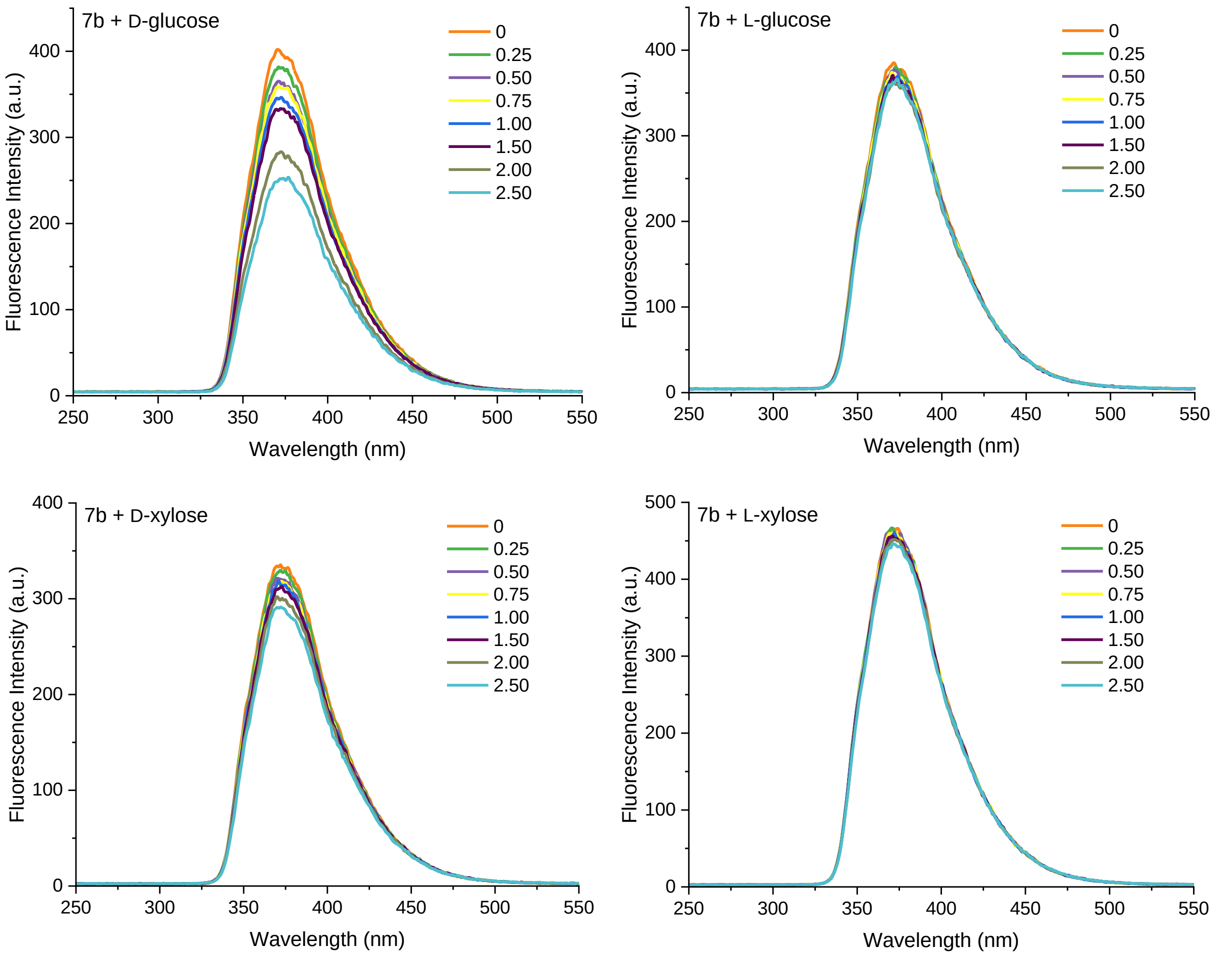

Figure S23. Fluorescence spectra of dyes **7b** ($4.43x10^{-5}$ M) in acetonitrile with different amounts (0-2.50 equiv.) of D- and L-glucose and D- and L-xylose in DMSO solution. (λ_{exc} = 305 nm, exc./em. slits 3.0/3.0 nm).

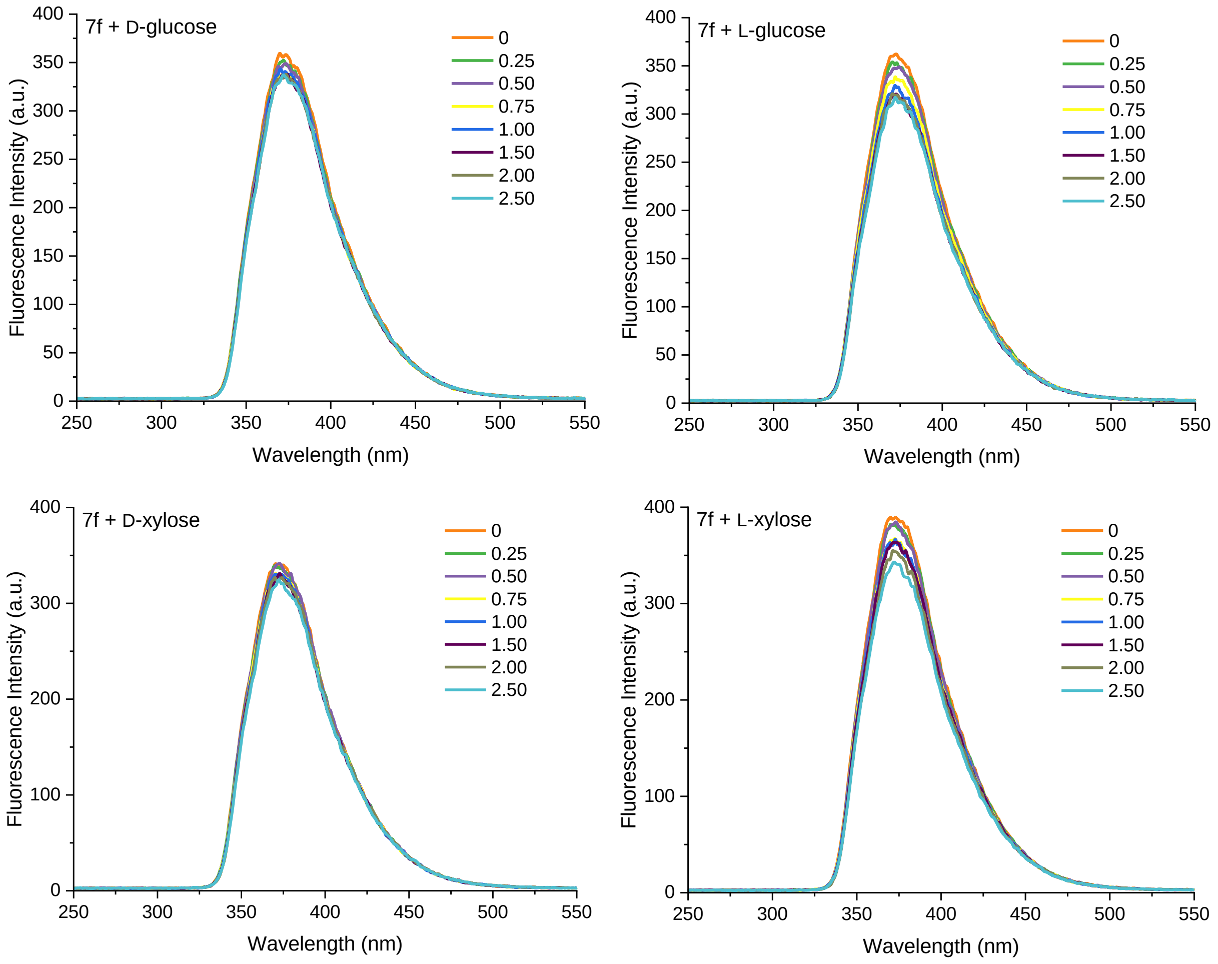

Figure S24. Fluorescence spectra of dyes **7f** ($3.35x10^{-5}$ M) in acetonitrile with different amounts (0-2.50 equiv.) of D- and L-glucose and D- and L-xylose in DMSO solution. (λ_{exc} = 305 nm, exc./em. slits 3.0/3.0 nm).

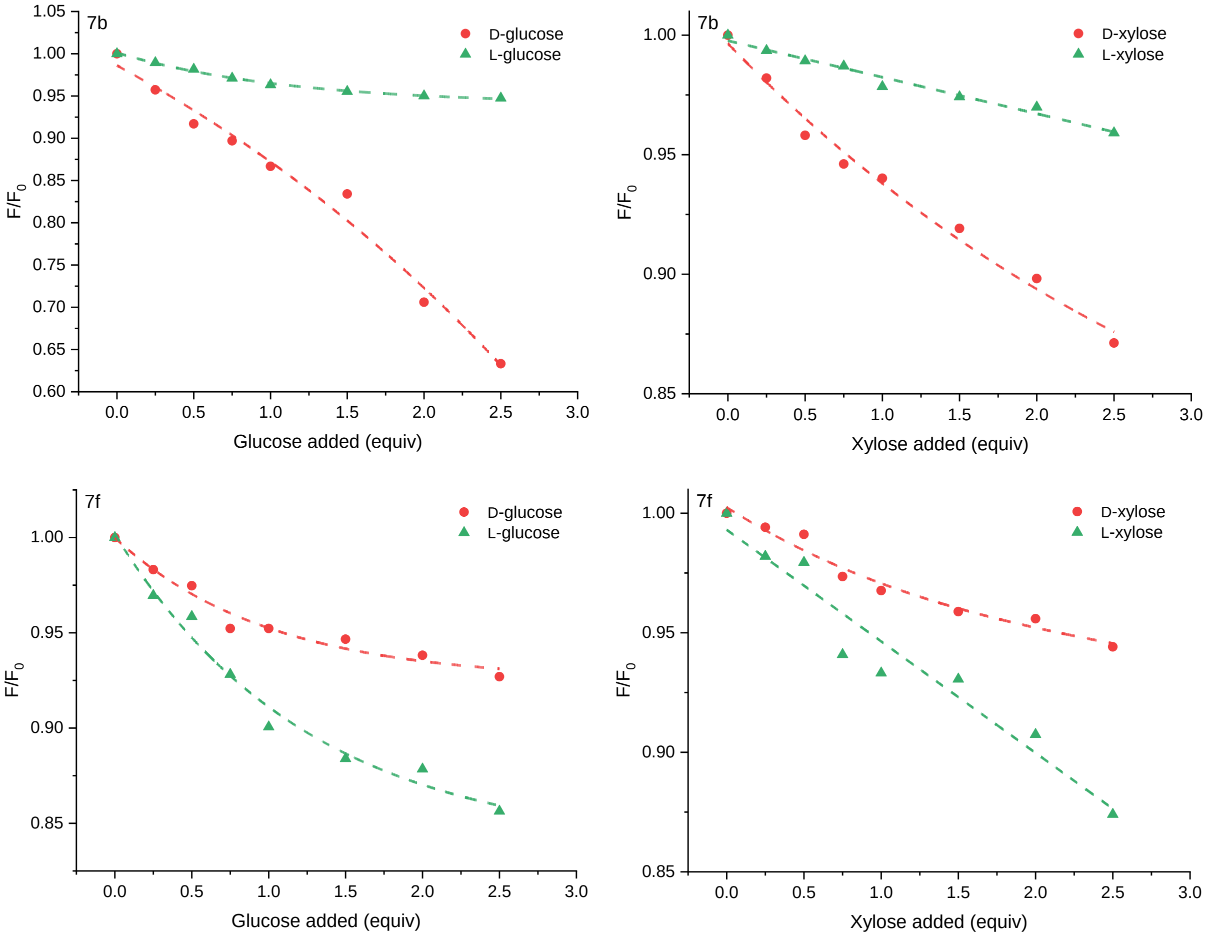

Figure S25. Graphical of the ratio of the fluorescence emission of dyes **7b** and **7f** *versus* different amounts (0-2.50 equiv.) of D- and L-glucose and D- and L-xylose at 305 nm.

8 Additional time-resolved fluorescence spectroscopy data

Analysis Function: quinta-feira outubro 24 2024 at 15:16

Decay curve: 7b puro

IRF curve: IRFnovo

Start Time: 60.35

End Time: 62.07

Offset will be calculated

Shift will be calculated

Pre-exp. 1: 1

Lifetime 1: 1

***** Statistics *****

Fitted curve: FLD Fit (66)

Residuals: FLD Residuals (66)

Autocorrelation: FLD Autocorrelation (66)

Deconvolved Fit: FLD Deconvoluted (66)

Chi^2: 0.9396

Durbin Watson: 2.051

Z: 0.007405

Pre-exp. 1: 4.962 ± 8.859e-002 (100 ± 1.785%)

Lifetime 1: 0.2351 ± 5.895e-003

F1: 1

Tau-av1: 0.2351

Tau-av2: 0.2351

Offset: 40.07

Shift: 0.0141

****

Analysis Function: quinta-feira outubro 24 2024 at 15:16

Decay curve: 7b_D_Ara

IRF curve: IRFnovo

Start Time: 60.35

End Time: 62.07

Offset will be calculated

Shift will be calculated

Pre-exp. 1: 1

Lifetime 1: 1

***** Statistics *****

Fitted curve: FLD Fit (67)

Residuals: FLD Residuals (67)

Autocorrelation: FLD Autocorrelation (67)

Deconvolved Fit: FLD Deconvoluted (67)

Chi^2: 1.042

Durbin Watson: 2.077

Z: -0.08195

Pre-exp. 1: 4.999 ± 1.351e-001 (100 ± 2.702%)

Lifetime 1: 0.2256 ± 7.908e-003

F1: 1

Tau-av1: 0.2256

Tau-av2: 0.2256

Offset: 58.95

Shift: 0.02393

****

Analysis Function: quinta-feira outubro 24 2024 at 15:16

Decay curve: 7b_L_Ara

IRF curve: IRFnovo

Start Time: 60.35

End Time: 62.07

Offset will be calculated

Shift will be calculated

Pre-exp. 1: 1

Lifetime 1: 1

***** Statistics *****

Fitted curve: FLD Fit (68)

Residuals: FLD Residuals (68)

Autocorrelation: FLD Autocorrelation (68)

Deconvolved Fit: FLD Deconvoluted (68)

Chi^2: 1.17

Durbin Watson: 1.461

Z: -0.2646

Pre-exp. 1: 4.933 ± 8.322e-002 (100 ± 1.687%)

Lifetime 1: 0.2293 ± 5.639e-003

F1: 1

Tau-av1: 0.2293

Tau-av2: 0.2293

Offset: 65.09

Shift: 0.05129

Table S2. Time-resolved fluorescence data in acetonitrile of sensor **7b** pure and in the presence of 2.5 equiv. of D- and L-Arabinose, where A is the preexponential factor, τ is the experimental fluorescence lifetime, and χ^2 determines the quality of the exponential fit.

System	A	τ (ns)	χ^2
**7b**	4.962	0.235	0.940
**7b**:D-Arabinose	4.999	0.226	1.042
**7b**:L-Arabinose	4.933	0.229	1.170

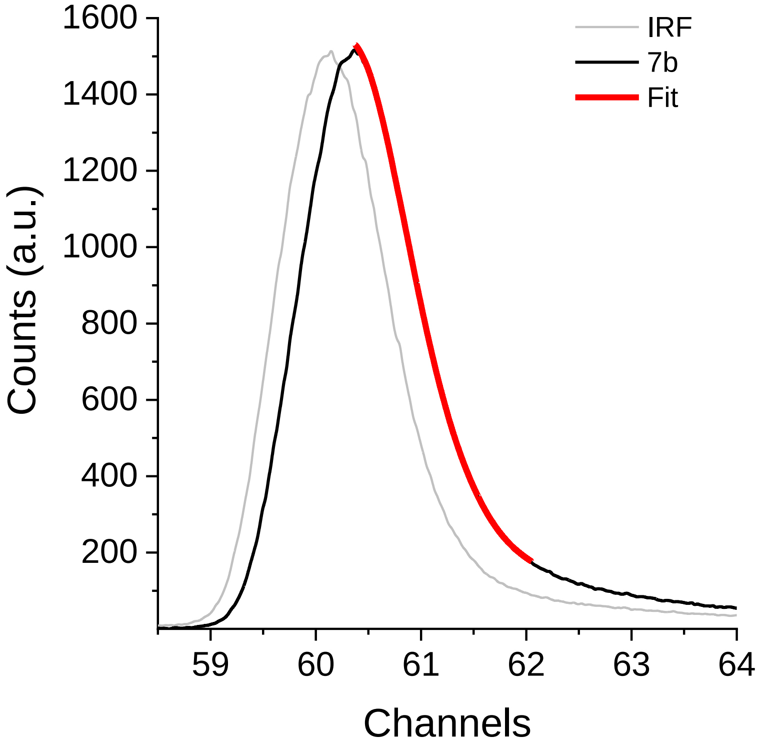

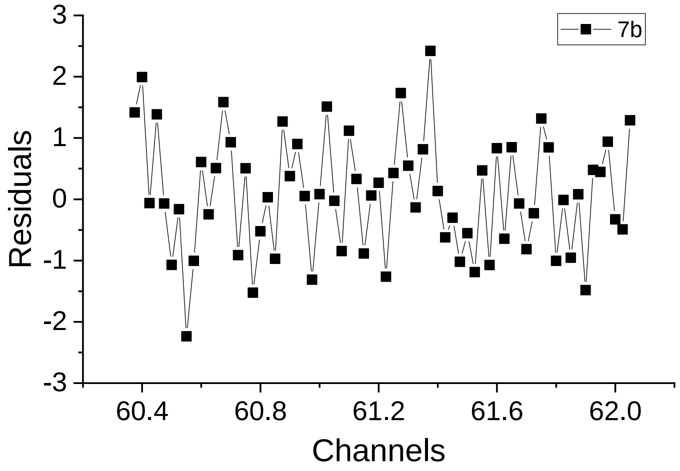

Figure S26. Time-resolved fluorescence decay (λ_{ex}=340 nm) of the compound **7b** in acetonitrile solution (5.23x10^{-5} M), IRF (instrument response factor), and respective exponential fit. Below: Residuals from the exponential fit.

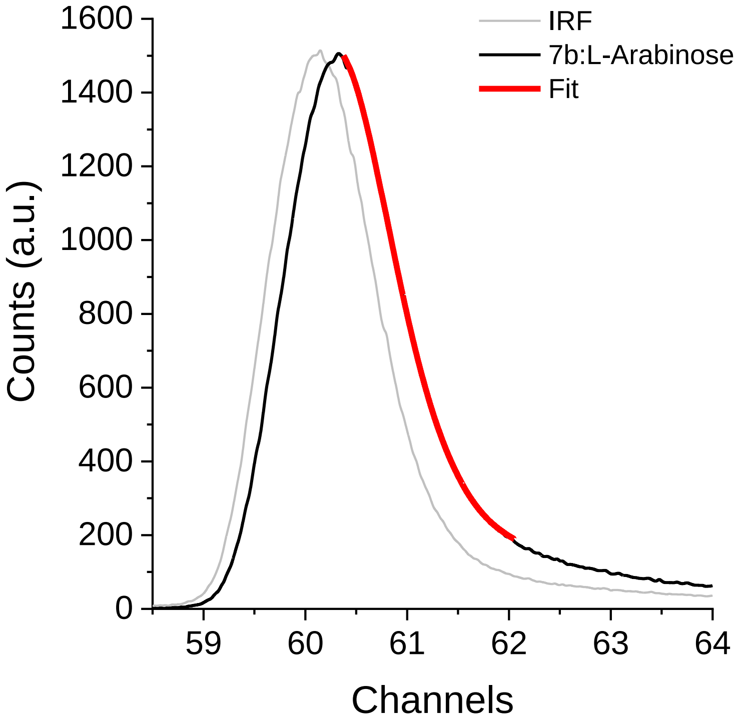

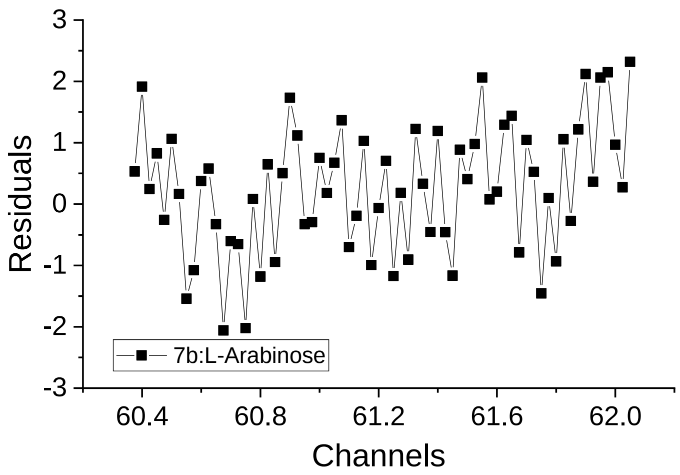

Figure S27. Time-resolved fluorescence decay (λ_{ex}=340 nm) of the compound **7b** in acetonitrile solution (5.23x10^{-5} M) in the presence of 2.5 equiv. of L-Arabinose, IRF (instrument response factor), and respective exponential fit. Below: Residuals from the exponential fit.

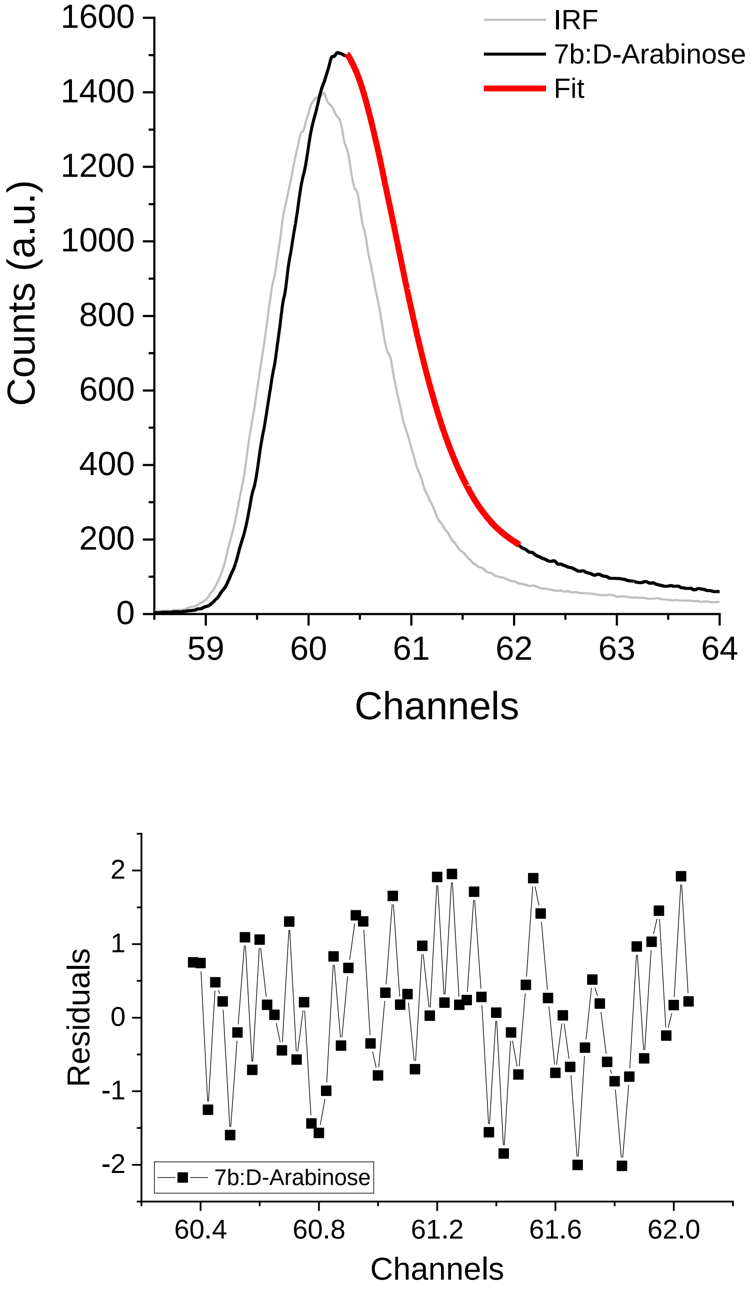

Figure S28. Time-resolved fluorescence decay (λ_{ex}=340 nm) of the compound **7b** in acetonitrile solution (5.23x10^{-5} M) in the presence of 2.5 equiv. of D-Arabinose, IRF (instrument response factor), and respective exponential fit. Below: Residuals from the exponential fit.

9 Additional computational data

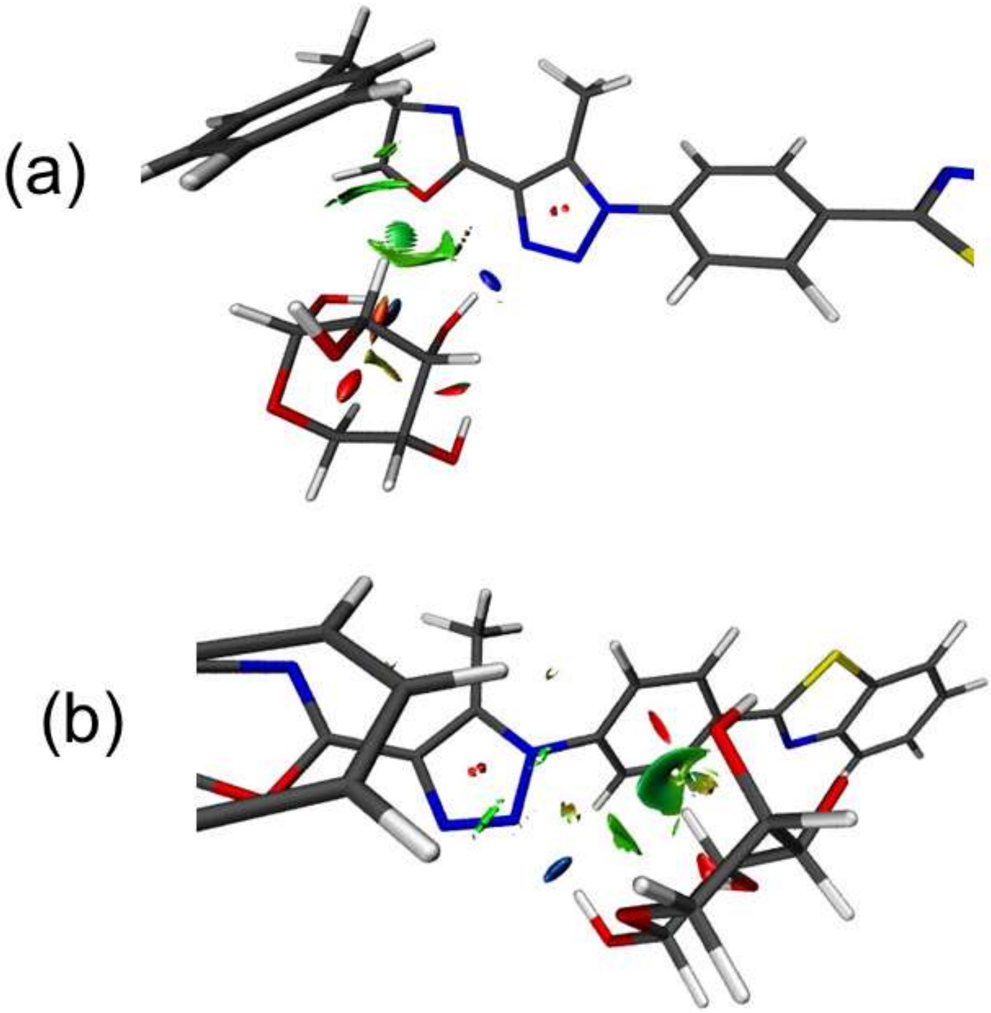

Figure S29. Interactions present between the **7b** with (a) D-arabinose and (b) L-arabinose. Green and blue regions represent Van der Waals interactions and hydrogen bonds, respectively.

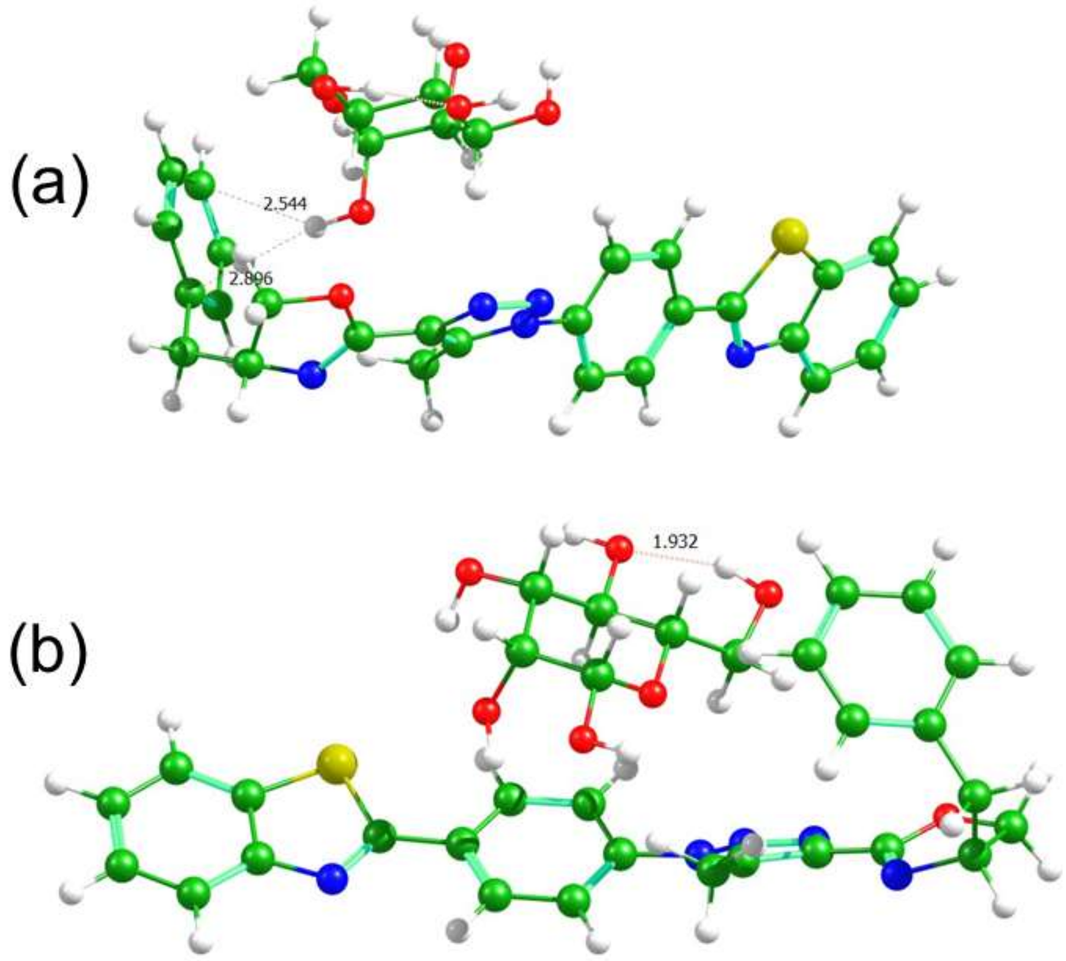

Figure S30. Interactions present between the **7b** with (a) D-manose and (b) L-manose. Green and blue regions represent Van der Waals interactions and hydrogen bonds, respectively.

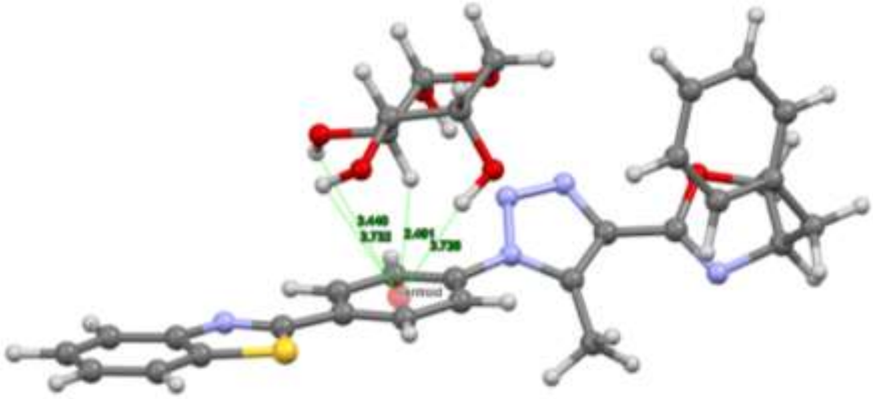

Figure S31. Distances obtained at the main site where a weak CH-π interaction exists between for the **7b** with L-arabinose.

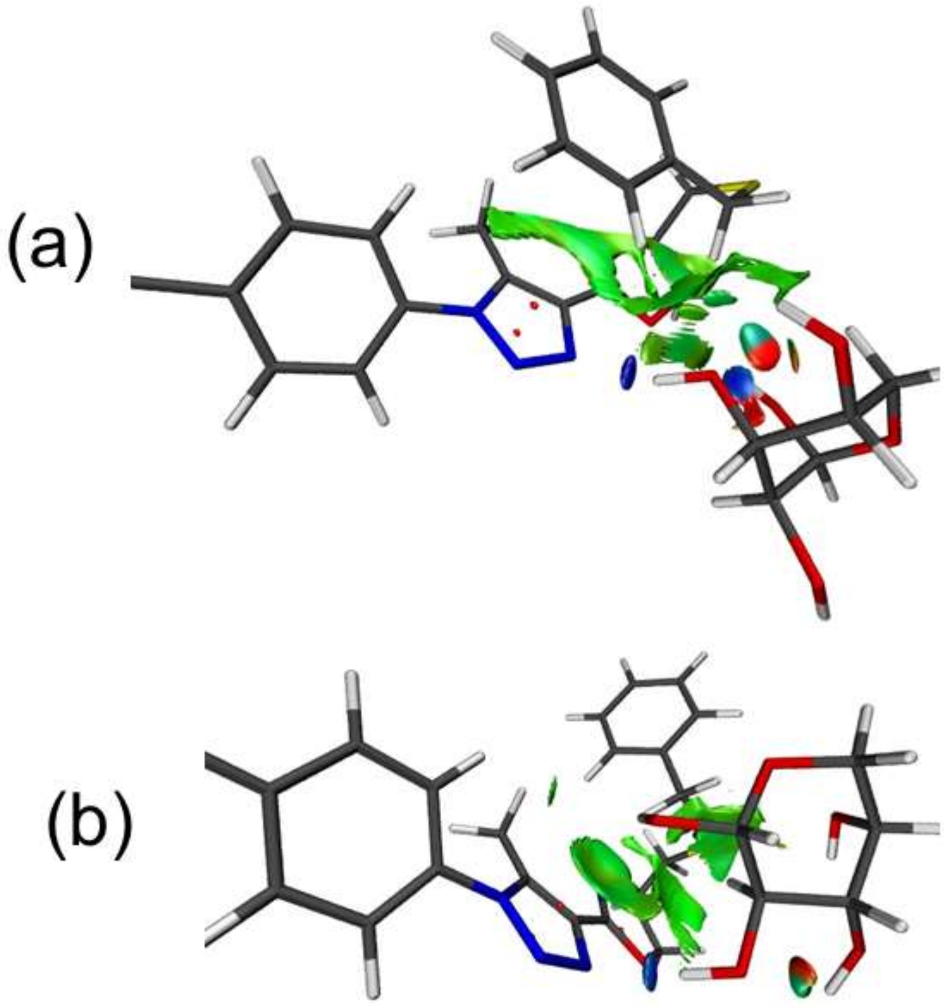

Figure S32. Interactions present between the **7f** with (a) D-arabinose and (b) L-arabinose (second). Green and blue regions represent Van der Waals interactions and hydrogen bonds, respectively.

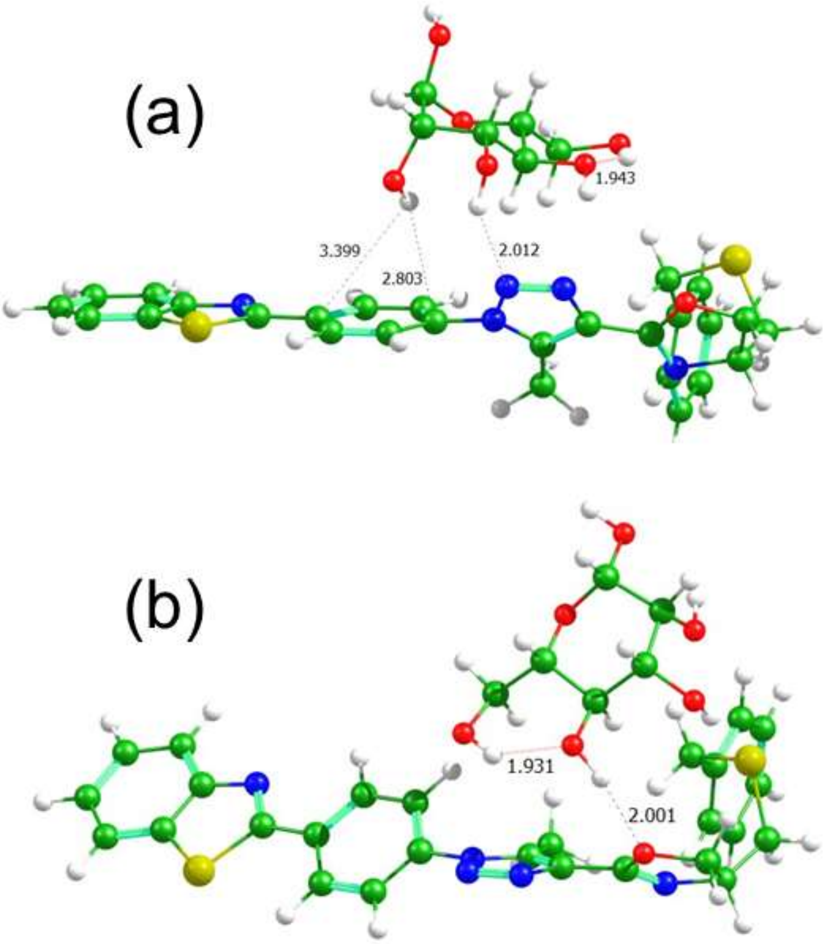

Figure S33. Interactions present between the **7f** with (a) D-manose and (b) L-manose. Green and blue regions represent Van der Waals interactions and hydrogen bonds, respectively.

---

[i] Still, W. C.; Kahn, M.; Mitra, A. Rapid Chromatographic Technique for Preparative Separations with Moderate Resolution. *J. Org. Chem*. **1978**, *43*, 2923-2925. DOI: 10.1021/jo00408a041.

[ii] Wurth, C.; Grabolle, M.; Pauli, J.; Spieles, M.; Resch-Genger, U. Relative and Absolute Determination of Fluorescence Quantum Yields of Transparent Samples. *Nat. Protoc*. **2013**, *8*, 1535-1550. DOI: 10.1038/nprot.2013.087.
